# Identification of Novel Cathepsin B Inhibitors with Implications in Alzheimer’s Disease: Computational Refining and Biochemical Evaluation

**DOI:** 10.3390/cells10081946

**Published:** 2021-07-31

**Authors:** Nitin Chitranshi, Ashutosh Kumar, Samran Sheriff, Veer Gupta, Angela Godinez, Danit Saks, Soumalya Sarkar, Ting Shen, Mehdi Mirzaei, Devaraj Basavarajappa, Morteza Abyadeh, Sachin K. Singh, Kamal Dua, Kam Y. J. Zhang, Stuart L. Graham, Vivek Gupta

**Affiliations:** 1 Faculty of Medicine, Health and Human Sciences, Macquarie University, F10A, 2 Technology Place, North Ryde, NSW 2109, Australia; samran.sheriff@hdr.mq.edu.au (S.S.); angela.godinez@hdr.mq.edu.au (A.G.); danit.saks@hdr.mq.edu.au (D.S.); soumalya.sarkar@hdr.mq.edu.au (S.S.); ting.shen@mq.edu.au (T.S.); mehdi.mirzaei@mq.edu.au (M.M.); devaraj.basavarajappa@mq.edu.au (D.B.); stuart.graham@mq.edu.au (S.L.G.); 2 Center for Biosystems Dynamics Research, Laboratory for Structural Bioinformatics, RIKEN, 1-7-22 Suehiro, Tsurumi, Yokohama 230-0045, Kanagawa, Japan; akumar@riken.jp (A.K.); kamzhang@riken.jp (K.Y.J.Z.); 3 School of Medicine, Faculty of Health, Deakin University, Geelong, VIC 3220, Australia; veer.gupta@deakin.edu.au; 4 Cell Science Research Center, Department of Molecular Systems Biology, Royan Institute for Stem Cell Biology and Technology, ACECR, Tehran 1665659911, Iran; mabyadeh@yahoo.com; 5 School of Pharmaceutical Sciences, Lovely Professional University, Phagwara 144411, Punjab, India; sachin.16030@lpu.co.in; 6 Discipline of Pharmacy, Graduate School of Health, University of Technology Sydney, Ultimo, NSW 2007, Australia; kamal.dua@uts.edu.au; 7 Australian Research Centre in Complementary and Integrative Medicine, Faculty of Health, University of Technology Sydney, Ultimo, NSW 2007, Australia

**Keywords:** Alzheimer’s disease, cathepsin B, 3D pharmacophore, virtual screening, docking, molecular dynamics

## Abstract

Amyloid precursor protein (APP), upon proteolytic degradation, forms aggregates of amyloid β (Aβ) and plaques in the brain, which are pathological hallmarks of Alzheimer’s disease (AD). Cathepsin B is a cysteine protease enzyme that catalyzes the proteolytic degradation of APP in the brain. Thus, cathepsin B inhibition is a crucial therapeutic aspect for the discovery of new anti-Alzheimer’s drugs. In this study, we have employed mixed-feature ligand-based virtual screening (LBVS) by integrating pharmacophore mapping, docking, and molecular dynamics to detect small, potent molecules that act as cathepsin B inhibitors. The LBVS model was generated by using hydrophobic (HY), hydrogen bond acceptor (HBA), and hydrogen bond donor (HBD) features, using a dataset of 24 known cathepsin B inhibitors of both natural and synthetic origins. A validated eight-feature pharmacophore hypothesis (Hypo III) was utilized to screen the Maybridge chemical database. The docking score, MM-PBSA, and MM-GBSA methodology was applied to prioritize the lead compounds as virtual screening hits. These compounds share a common amide scaffold, and showed important interactions with Gln23, Cys29, His110, His111, Glu122, His199, and Trp221. The identified inhibitors were further evaluated for cathepsin-B-inhibitory activity. Our study suggests that pyridine, acetamide, and benzohydrazide compounds could be used as a starting point for the development of novel therapeutics.

## 1. Introduction

Alzheimer’s disease (AD) is the most common type of dementia [1], associated with decline in memory and cognition [2,3]. AD is the sixth-leading cause of death, with one-third of senior citizen deaths resulting from AD [4]. In its advanced stages, AD is characterized by the reduction of cerebrospinal fluid amyloid β (CSF Aβ), and enhanced accumulation of Aβ in the brain and the retinas [4,5,6]. Brain atrophy and toxicity associated with Aβ aggregation are key features of the disease in the vast majority of patients [7]. AD and other forms of dementia are caused by damage to neurons, and most of the current drugs have failed to meet the primary endpoints in phase 3 clinical trials. Therefore, the disease remains open to potential genetic and pharmacological therapies which, in addition to the strategy of clearance of Aβ aggregates, include therapies to slow down or prevent the loss of brain neurons and maintain their function [8,9].

Cathepsin B (EC 3.4.22.1), belongs to the papain-like cysteine protease family, and displays carboxyl peptidase activity involving the degradation of proteins and peptides [10]. The active form of this enzyme [11] mediates extracellular matrix degradation in different pathological conditions, including myocardial infarction [12], cancer [13], pancreatitis [14], and AD [15]. It is important to note that there is an increase in plasma levels of cathepsin B in the abovementioned conditions. Interestingly, the other isoforms of cathepsin (S, V, and K) are found to be localized in specific tissues, with unique biological functions [16,17,18,19].

The autosomal-dominant mutations in AD have been identified in three genes: *APP, presenilin 1 (PSEN1),* and *presenilin 2 (PSEN2)* [20]. The presenilins are components of the proteolytic γ-secretase complex that, together with β-secretase, generates Aβ. Recently, our studies on single-nucleotide polymorphisms (SNPs) associated with the cathepsin B gene have provided relevant insight into functional and haplotype tag SNPs related to protein functions in AD [21]. Previous studies on cathepsin B protein inhibition revealed that most of its inhibitors can bind to the target protein irreversibly, while some bind reversibly [22]. The irreversible inhibitors include dipeptidyl nitriles [23], fluoromethyl ketones [24], vinyl sulfones [25], expoxysuccinates [26], and peptidyl epoxides [27]. The reversible inhibitors include peptidyl aldehydes [28] and glyoxal. [29]. The inhibitory mechanism of epoxysuccinyl, dipeptidyl nitriles, hydroxycarboxyethyl-carbonyl and ethoxy-carbonyl compounds on cathepsin B enzyme catalytic activity has been reported previously [23,30,31]. Cathepsin B inhibitors from natural sources have previously demonstrated cathepsin B enzyme inhibitory activity in vitro in the range of 0.62–1.17 μM [32].

**Figure 1:**
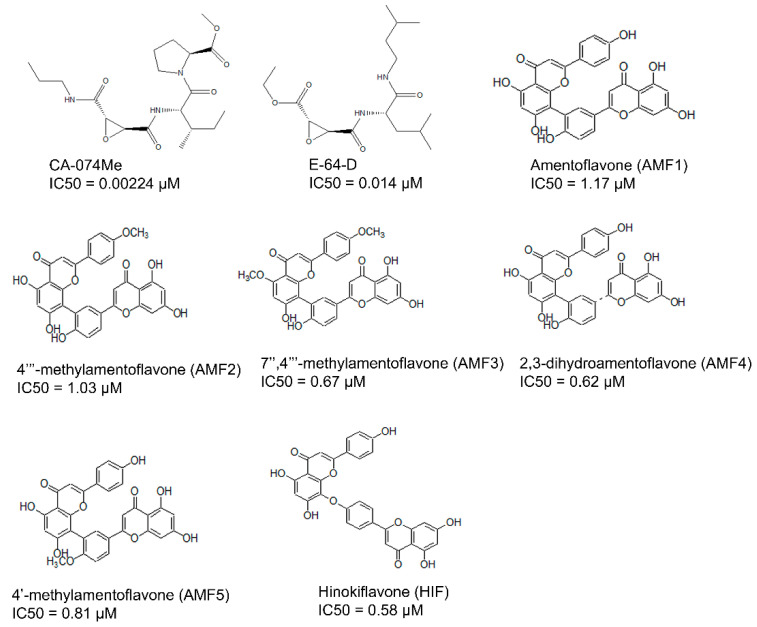
Previously reported inhibitors of cathepsin B, with IC50 in µM.

These studies indicate cathepsin B as an attractive target for drug development for several diseases, especially AD. However, until now, only a few cathepsin B inhibitors have been reported (Figure 1) [32]. Therefore, it is imperative to identify small drug molecules that are safe and suitable for chemical optimization and development into therapeutic agents to target AD. Motivated by the need for chemical scaffolds for the discovery of novel of cathepsin B inhibitors, we carried out ligand-based virtual screening, molecular docking, and dynamics studies. In this study, we generated mixed-feature three-dimensional pharmacophore models from the cathepsin B inhibitors (synthetic and natural origin). We employed a large-scale virtual screening of the Maybridge database library, consisting of ~61,000 small molecules. Furthermore, the selection of several filters was applied for the identification of novel inhibitors against cathepsin B. We employed protein–ligand interactions and made use of fingerprint clustering techniques to prioritize virtually screened hits and identify cathepsin B inhibitors. Furthermore, to validate the stability of the lead molecules to close the occluding loop, the docked poses with the highest proximity stability indicated by their low root-mean-square deviations (RMSDs) were subjected to molecular dynamics studies for a 20-ns timeframe.

Our step-by-step methodology provided a fast-track approach to screening large databases. The three key hit molecules identified in the study—BTB03075, KM02922, and RF02795—can be utilized in the future design and characterization of cathepsin B inhibitors.

## 2. Materials and Methods

### 2.1. Selection and Preparation of Macromolecule

The crystal structure of the cathepsin B protein (PDB ID: 1CSB) was retrieved from the Protein Data Bank (PDB) (http://www.pdb.org, accessed on 14 April 2021) [33,34]. The protein macromolecule was prepared to calculate the binding energy. The polar hydrogens were added to the molecule, whereas the nonpolar hydrogens were removed from the protein coordinates, and the Gasteiger partial charges were subsequently added to the carbon atom containing hydrogens [35]. Protein optimization was carried out with UCSF Chimera software [36], under AMBER parameters, and followed by protein minimization using the MMTK method in 500 steps, with a step size of 0.02 Å. The protein molecule contains light chains A and D, with 47 amino acids, and heavy chains B and E, including 205 amino acids, with a resolution of 2.00 Å. Active site residues of the binding pocket were validated from previous reports [10].

### 2.2. Collection and Preparation of Ligand Dataset

**Figure 2:**
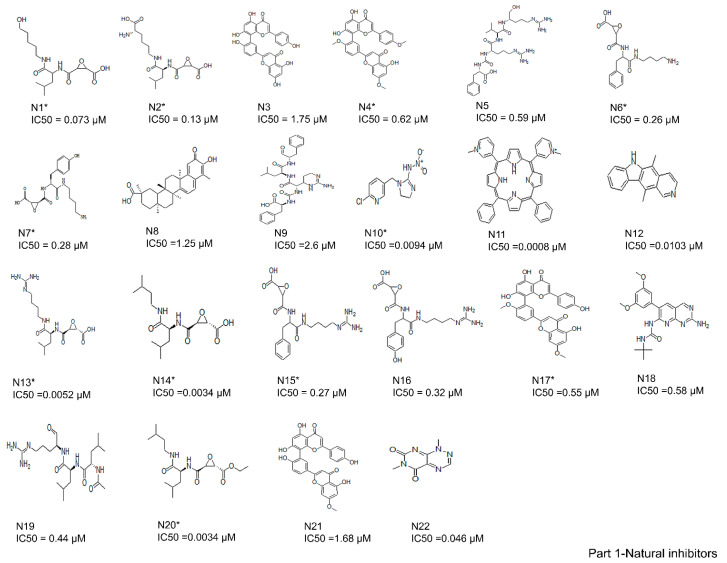

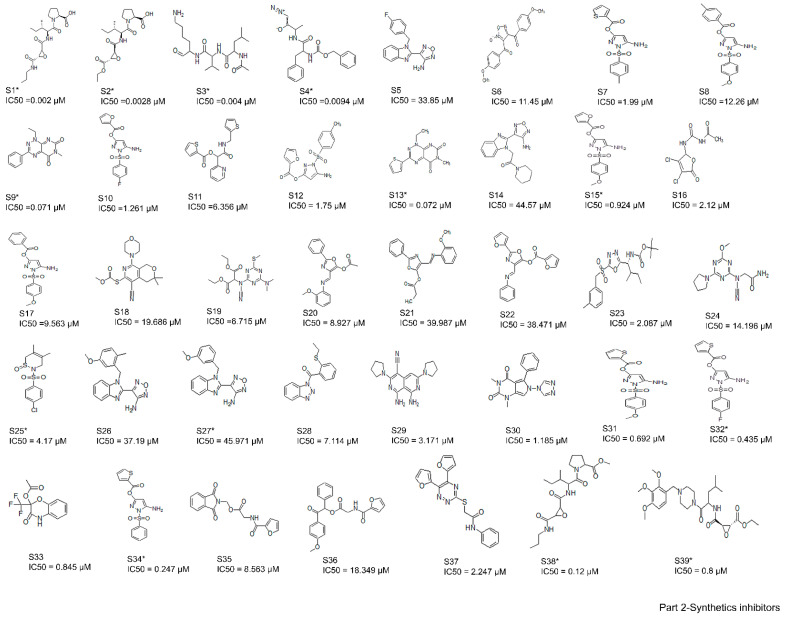
Two-dimensional structures of compound datasets from natural origin (Part 1) and synthetic origin (Part 2). The compound numbers and IC50 values are shown at the bottom of the respective compounds. The training sets are denoted with asterisks.

A total of 61 analogs of the inhibitors—22 of natural (N) (Figure 2-part 1) and 39 of synthetic (S) (Figure 2-part 2) origin—were collected from the PubChem database [37] and other literature [26,38,39,40]. Inhibitors were screened based on their pharmacological properties (IC50 and Ki values), as shown in Figure 2. The qualitative and quantitative characterization—such as physicochemical properties—was generated using the Molinspiration web server (http://www.molinspiration.com, accessed on 16 April 2021). The absorption, distribution, and toxicity prediction are summarized in Tables S1–S3, respectively. Inhibitor optimization was carried out using Sybyl-x1.1 with a conjugate gradient method (TRIPOS Inc.1699, St. Louis, MO, USA).

### 2.3. Molecular Docking and Interaction Studies

Molecular docking of 61 analog inhibitors was carried out using a Lamarckian genetic algorithm (LGA) with AutoDock v4.2 tools with default docking parameters [41]. The receptor file was first prepared by deleting water molecules and adding polar hydrogens only. Gasteiger charges and any other atoms present were assigned AD4 atom types. Ligands with rotatable bonds were set to free, and the protonation state was maintained at physiological pH [35]. Grid parameter files were built, and atom-specific three-dimensional affinity maps were constructed using AutoGrid v4.2. The three docking steps (van der Waals interactions, hydrogen bonds, and the electrostatic potential) were considered for the calculation of binding energy [42,43]. Each docking experiment was derived from 20 different runs that were set to terminate after a maximum of 2,500,000 energy evaluations, or 27,000 generations, yielding 20 docked conformations, while the population size was set to 150. After the generation of multiple runs, cluster analysis was performed. The docking solutions with inhibitors that had atomic root-mean-square deviations (RMSDs) within 2.0 Å of one another were clustered together and ranked by their lowest energy. The solution with the lowest energy was accepted as the best docking conformation for the binding energy calculation. Estimated binding free energy (kcal/mol), inhibitory constant (Ki, µM), electrostatic energy (kcal/mol), van der Waals interactions (kcal/mol), hydrogen bonds (kcal/mol), desolvation energy (kcal/mol), total intermolecular energy (kcal/mol), torsional energy (kcal/mol), and Gibbs free energy of binding were calculated for 61 cathepsin B inhibitors.

### 2.4. Ligand-Based Three-Dimensional Pharmacophore Search and Virtual Screening

A ligand-based three-dimensional pharmacophore model based on the chemical features present in 61 active, moderately active, and inactive cathepsin B inhibitors was generated using LigandScout 3.1 software (Inte:Ligand GmbH, v3.1, Vienna, Austria) [44] from the docking of 61 cathepsin B inhibitors, so that these ligands (cathepsin B inhibitors) would be flexibly aligned into a rigid macromolecular (cathepsin B protein) environment, and later could be estimated for the fidelity of the interactions [45]. Pharmacophoric descriptors—including H-bond donors (HBD), H-bond acceptors (HBA), hydrophobic (HY), ring aromatic (RA), positive ionizable (PI), and negative ionizable (NI) features—were mapped onto the chemical features of all training set molecules [42]. RA, PI, and NI features were less significantly mapped when compared to the other features. Therefore, HBD, HBA, and HY were selected to generate 3D pharmacophore hypothesis protocols. The docked pose of each compound in the training set was used to generate maximum numbers of conformations (500), a root-mean-square (rms) threshold of 0.4, energy window of 10, and maximum pool of 4000 as the default values using the ligandset module in LigandScout 3.1. Furthermore, clustering was performed using similarity measurement via pharmacophore alignment score. The cluster distance calculation method was kept at 0.4 and average in order to generate ligand-based pharmacophores using pharmacophore-fit and atom overlap scoring functions. All of the other parameters were set as default. The IC50 values of individual training set compounds were selected as active properties. The test set analysis method was used to validate the pharmacophore model hypothesis. This method was validated by the enrichment factor (E) to discriminate between active and inactive molecules, and expressed as:

E = Ha × D/Ht × A(1)

False negative (A−Ha)(2)

False positive (Ht−Ha)(3)

Goodness of hit score (GH)(4)

The enrichment factor was calculated as the ratio of active hits (Ha) amongst all the molecules in database D and total hits (Ht) in database A (Equation (1)). The false negatives were calculated by subtracting active hits (Ha) from the total number in the database (A) (Equation (2)), and the difference in total hits (Ht) and active hits (Ha) was used to calculate the rate of false positives (Equation (3)). The goodness of hit score (GH) ranged from 0 to 1, differentiating null models from ideal models (Equation (4)).

### 2.5. Screening of Maybridge Database and Fast Docking Using AutoDock Vina

To identify potential lead molecules from the large Maybridge database containing 60,538 small molecules, virtual screening was performed by generating ligand-based three-dimensional pharmacophores (as described in the previous section). At the start of the virtual screening, we presumed that possible hit molecules from the Maybridge library should fit with the majority of the possible features of the pharmacophore query. Duplicate Maybridge molecules were removed and not used for screening. Thereafter, the Maybridge-screened compound hits were docked into the binding site of cathepsin B using AutoDock Vina (ADt-Vina) [46]—a fast-docking program to remove compounds with non-compatible geometries and energetics toward the ligand-binding site. ADt-Vina used an “iterated local search global optimizer” algorithm to evaluate the interactions between a ligand and its receptor. The search space for docking was restricted to a cubic box of 40 Å × 40 Å × 40 Å centered on the binding site. ADt-Vina calculations were carried out using a default value of eight to ensure the exhaustiveness of the global search. Twenty poses were generated for each ligand, which were ranked by ADt-Vina’s empirical scoring functions, and the pose with the lowest docking score value was selected as the best iteration. The predicted binding energy from the docking provided a ranking of the compounds, which was compared to the known actives using two measures. Virtual screening performance is commonly analyzed using a receiver operating characteristic (ROC) curve, which can easily be quantified by determining the area under the curve (AUC). The AUC and the Boltzmann-enhanced discrimination of receiver operating characteristic (BEDROC) metric were used to evaluate the robustness of the docking algorithm in order to select active compounds [47]. The identified hit molecules were further subjected to various screening filters, such as Lipinski’s rule of five, which selects molecules with molecular weight less than 500 D, HBD less than 5, HBA less than 10, and a LogP (octanol/water partition coefficient) value of less than 5 [48] to achieve the possible set of potential cathepsin B inhibitor molecules.

### 2.6. Construction of Structural Interaction Fingerprints (SIFts), Similarity Analysis, and Hierarchical Clustering

Structural interaction fingerprints (SIFts) were used for one-dimensional binary representation of contact between ligands and macromolecule amino acids. The advantage of using the SIFt method is in generating protein–ligand interaction fingerprints to elucidate the interaction patterns [49,50,51]. Predefined binding site amino acid residues involved in ligand binding were used for SIFt calculations for both sets of ligands (inhibitors of cathepsin B and virtual screen hits), while hydrogen bond interactions between ligands and amino acids were identified by UCSF Chimera software with default settings. The next step was to classify protein–ligand interaction fingerprints. The implementation of SIFt uses seven bits for each amino acid residue-contacting ligand. The seven bits were switched on (binary representation: 1) and off (binary representation: 0) depending on several factors, such as (1) the subsequent interactions specifying whether a contact was involved at a position, whether the (2) main-chain (MC) or (3) side-chain (SC) atoms were involved, and the presence of a (4) hydrogen bond acceptor (HBA) or (5) donor (HBD); the (6) polar and (7) nonpolar nature of the interactions thus represent each residue by a seven-bit-long string. The complete interaction fingerprints of the virtual screen molecules that complexed with the proteins were finally constructed by successively concatenating the bit string of each binding site residue simultaneously, in accordance with the ascendant residue number. This results in each interaction fingerprint being similar in length, and enables easy comparison of protein–ligand binding interactions at a binding site position across a series of complexes.

For the quantitative measure of bit string similarity, we used the Jaccard–Tanimoto coefficient (JT), which is widely used for binary data [52]. The RTc between the two strings A and B is calculated as:

$$\mathrm{JT}\;(A,\;B)=\frac{\left|A\cap B\right|}{\left|A\cup B\right|}$$

where |A∩B| represents the number of ON bits common in both A and B, and |A∪B| represents the number of ON bits present in either A or B. The SIFt signifies the protein–ligand interaction pattern; however, similarity analysis of the fingerprints implies that the ligands carry out similar interactions with the protein. We then applied an algorithm that groups similar objects into groups of similar clusters—hierarchical clustering—in order to analyze the protein–ligand interaction fingerprints for each test case [53], using SYSTAT v13.2 software. The generated protein–ligand complex clusters were manually inspected based on their fingerprint interaction dendrograms.

### 2.7. Molecular Dynamics Simulation and Binding Free Energy Calculations

The molecular mechanics Poisson−Boltzmann surface area (MM-PBSA) [54,55] and molecular-mechanics-generalized Born surface area (MM-GBSA) [56] methods, as implemented in AMBER10 [57], were used to calculate ligand binding free energy. To generate a conformational ensemble for the binding free energy calculation, molecular dynamics simulation was carried out for compounds selected after docking. In each case, the proteins were immersed in the rectangular truncated octahedron filled with 8 Å TIP3P water molecules and neutralized by adding Na^+^ or Cl^−^ ions. At first, the protein system was minimized by 500 steps of steepest descent, followed by 2000 steps of conjugate gradient. After minimization, the system was gradually heated in the canonical ensemble from 0 to 300 K over 50 ps, and then equilibrated for 200 ps. Finally, a 20-ns MD simulation was performed under a constant temperature of 300 K [58]. The long-range electrostatic interactions were dealt with by employing the particle mesh Ewald (PME) method [59]. All hydrogen atoms were constrained using the SHAKE algorithm [60], and the time step was set to 2 fs. All simulations were performed using the SANDER module of AMBER10 [61] with the AMBER force field (ff03). Binding free energy was calculated using the following equation:

ΔGbinding = Gcomplex − (Gprotein + Gligand) (5)

The above equation can be conceptually summarized as:

ΔGbinding = ΔGMM + ΔGpolar solvation + ΔGnonpolar slovation − TΔS(6)

ΔGMM = ΔEvdw + ΔEelec + ΔEint (7)

where ΔGMM is the molecular mechanics binding free energy between small molecules and cathepsin B. The ΔEvdw, ΔEelec, and ΔEint account for differences in van der Waals energy, electrostatic energy, and internal energy, respectively. ΔGpolar solvation is the polar contribution to solvation-free energy, and is computed by solving the Poisson−Boltzmann equation in MM-PBSA, or by using Onufriev’s generalized Born model [62] in MM-GBSA. ΔGnonpolar solvation is the nonpolar contribution to solvation-free energy, and is estimated using the following equation:

ΔG_nonpolar slovation_ = γSASA + b (8)

where SASA is the solvent’s accessible surface area, as calculated using the LCPO method [63] for MM-PBSA and the ICOSA method [63] for MM-GBSA. The γ and b are empirical constants with default values of 0.0072 and 0, respectively. The TΔS represents the solute entropy that was not considered in this study, as we were mainly interested in the relative ranking of virtual screening hits, rather than absolute binding free energies.

### 2.8. Cathepsin B Inhibitory Assay

Cathepsin B inhibitory assay was performed using a fluorometric-based assay kit as per company instructions (ab185438). The assay kit utilizes the ability of cathepsin B to cleave synthetic AFC (7-Amino-4-trifluoromethyl coumarin) substrate to freely release AFC; moreover, this AFC can be quantified by a fluorometer. In the presence of cathepsin B inhibitors, the cleavage of the substrate was reduced, and there was a decrease in the total loss of the AFC fluorescence. The experiments were performed in triplicate. The test compounds (virtual screened hits) were prepared in different concentrations of 2.5, 5.0, 10.0, 15.0, and 20.0 µM. The inhibitor screening protocol was performed by first incubating 10 µL of test compound with cathepsin B enzyme in CTSB (cathepsin B) reaction buffer for 10–15 min at room temperature. one microliter of F-F-FMK (CTSB inhibitor) and 9 µL of CTSB reaction buffer were added to the cathepsin B enzyme well plate and treated as inhibitory controls (IC or positive control), whereas solvent with cathepsin B enzyme was used for enzyme control (EC or negative control). After incubation, 40 µL of cathepsin B substrate solution was added into each well and mixed. Next, fluorescence was measured in a kinetic mode for 30–60 min at 37 °C (Ex/Em = 400/505). Two timepoints were used—0 and 15 min (ΔT = T2 − T1)—and corresponding values for the fluorescence (ΔRFU = RFU2-RFU1) were used to calculate the slope for all test inhibitor samples and EC, by dividing the net ΔRFU values by the time ΔT. The percentage of relative inhibition was calculated using the following formula:

$$\%\;\mathrm{Relative}\;\mathrm{Inhibition}=\frac{\mathrm{Slope}\;\mathrm{of}\;\mathrm{EC}-\mathrm{Slope}\;\mathrm{of}\;\mathrm{Sample}\;}{\mathrm{Slope}\;\mathrm{of}\;\mathrm{EC}}\times 100$$

## 3. Results

**Figure 3:**
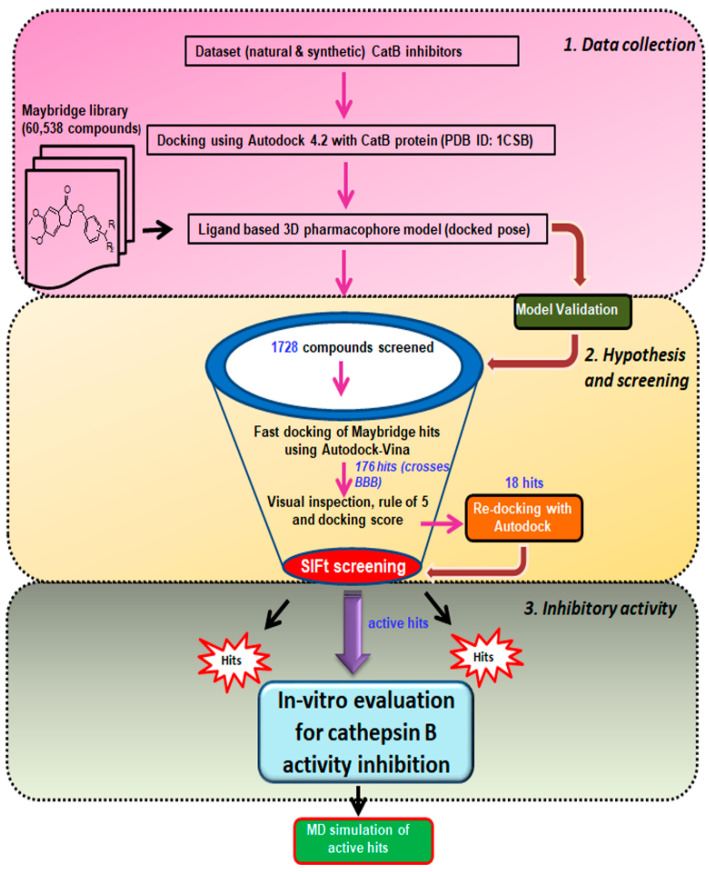
Schematic representation of ligand-based virtual screening of cathepsin B inhibitors. The study was performed in 3 different steps: Step 1, data collection: The dataset of 61 ligands was docked in cathepsin B enzyme (PDBID: 1CSB), and then used to generate a 3D pharmacophore, followed by screening of the Maybridge library. Step 2, hypothesis, and screening: Maybridge hits were subjected to fast docking using AutoDock-Vina, screening using the structural interaction fingerprint (SIFt) approach, and re-docking of the Maybridge virtual screen hits. Step 3, inhibitory activity: Biological evaluation of virtual screening hits against cathepsin B inhibitory activity and molecular dynamics; MM-PBSA and MM-GBSA were used to predict binding free energy components.

The current study employed the power of a computational approach to generate a three-dimensional pharmacophore hypothesis, refine the pharmacophore model through structural interaction fingerprinting, and validate the novel hits from the Maybridge database using molecular dynamics. The general strategy for the ligand-based pharmacophore virtual screening is presented in Figure 3. In summary, the 61 known cathepsin B inhibitors were docked at cathepsin B protein (PDB ID: 1CSB) active sites. The lowest binding energy interactions were selected to create ligand-based three-dimensional pharmacophore models. The validated Hypo III was utilized for the virtual screening of the Maybridge database. The Maybridge database of small molecules with diverse drug-like structures—containing 60,538 compounds—was screened within the matching features of the pharmacophore models. To our satisfaction, 1728 Maybridge molecules were closely mapped on all pharmacophoric features present in Hypo III. These molecules were additionally examined by subjecting them to fast-track docking using the ADt-Vina program, which provided 176 molecules with cutoff values of docking scores ≥ −6.0 kcal/mol. Furthermore, Lipinski’s rule of five was applied to 176 molecules as an additional parameter, which provided 18 molecules. These 18 hits were finally subjected to re-docking, and structural interaction fingerprints were generated to prioritize and refine the drug-like compounds. Three novel hit compounds were selected for molecular dynamics simulation studies and validated for further in vitro activity using enzyme-based assay methods. A schematic representation of the pharmacophore generation and virtual screening processes is shown in Figure 3.

### 3.1. Binding Modes of the 61 Active Cathepsin B Inhibitors Using the AutoDock v4.2 Docking Program

**Figure 4:**
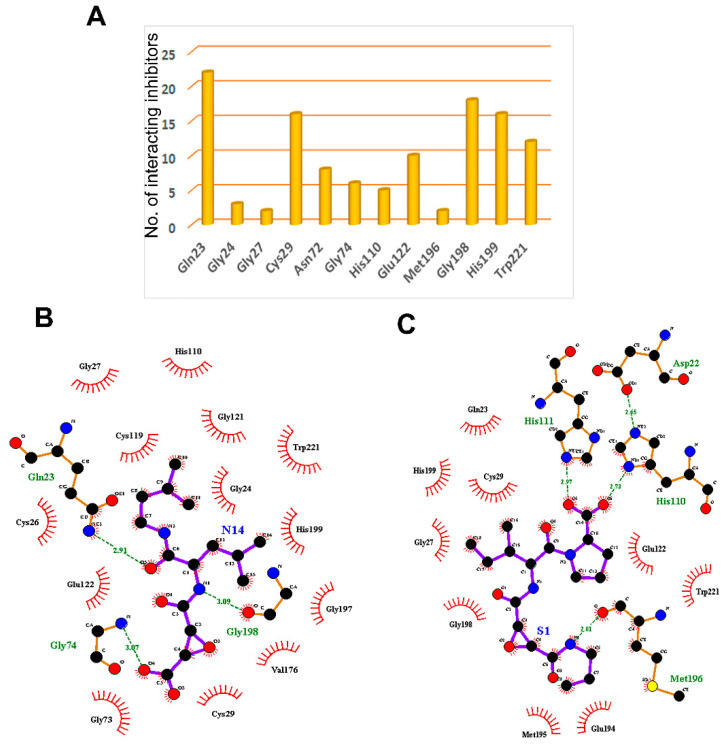
The key residues of the cathepsin B protein involved in interaction studies. (**A**) Dataset of 61 known cathepsin B inhibitors. LigPlot+ (EMBL-EBI, v2.2, Hinxton, Cambridge, UK) analysis results represent two-dimensional protein–ligand interactions for highly active compounds of (**B**) natural and (**C**) synthetic origins. Hydrogen bonds are shown in green dotted lines, while residues interacting by hydrophobic interactions are represented in red.

Molecular docking not only provides a visualization of potential binding orientations, but also the iteration by which the ligand interfaces with the relevant amino acid [64]. The hydrogen-bonding interaction was observed with critical residues such as Cys29 and Gly74. Traditional protein–ligand docking was performed with AutoDock v4.2 for the dataset of 61 cathepsin B inhibitors using LGA. The docking results predicted binding energies, inhibitory constants, and interactions, which have been calculated for each of them (Table 1 and Figure 4A). Gln23, Gly24, Gly27, Cys29, Asn72, Gly74, His110, Glu122, Met196, Gly198, His199, and Trp221 amino residues comprise the cathepsin B protein-binding site. One example each from both the natural and synthetic cathepsin B inhibitors with the lowest IC50 and binding energies has been selected here to explain the binding mode of these inhibitors into the cathepsin B protein. The natural molecule compound N14, containing an epoxide moiety, was oriented downwards, forming hydrogen bounds with Gln23, Gly74, and Gly198. As shown in Figure 4B, the oxygen atom of the C-5 carboxyl group and C-6 hydroxyl group of ligand-N14 is coordinated with Gln23 and Gly74m whereas the nitrogen atom of ligand-N14—the N-1 amine group—coordinated with Gly198 within the enzyme’s catalytic site. However, the synthetic class of inhibitors—compound S1—with the same epoxide moiety analogue, showed a different pattern of hydrogen-binding scheme. For this class of compounds, amino acids His110, His111, and Met196 were the key residues, with distance constraints of 2.73 Å, 2.97 Å, and 2.81 Å, respectively. The pyrrolidine substituent at the side chain of the S1 inhibitor involved the oxygen atom; the C-14 carboxyl group coordinated with His110 and His111, whereas the nitrogen atom of ligand-S1—the N-2 amine group—was found to coordinate with Met196 within the enzyme catalytic site (Figure 4C).

### 3.2. Generation and Validation of Mixed-Feature Ligand-Based Pharmacophore Models Using LigandScout 3.1

In total, 61 compounds were identified from various literature and, as described previously, 24 of them were selected manually, considering the structural diversity and wide range of activity, as the training set (marked with * in Figure 2), while the remaining compounds were used as the test set. Structures and biological activities of the training and test set compounds are indicated in Figure 2. The training and test set compounds were divided based on distribution of biological activities and chemical features. The dataset was classified into three categories, according to biological activity data: active (+++, IC50 ≤ 0.3 µM), moderately active (++, 0.3 µM < IC50 < 2.5 µM), and inactive (+, IC50 ≥ 2.5 µM). These molecules were distributed between the training set and test set. A maximum number of active and moderately active compounds, along with a few inactive compounds, were assigned to the training set compounds. The rest of the molecules were assigned to the test set for validation. The training set molecules included epoxides, pyridine, pyrrolidine, and potent acetamide compounds with high affinity to inhibit the cathepsin B domain. These available compounds provide ideal chemical structures for the development of cathepsin B inhibitors. Thus, these mixed-features pharmacophores were used to generate hypotheses based on the activity value of training set compounds.

**Figure 5:**
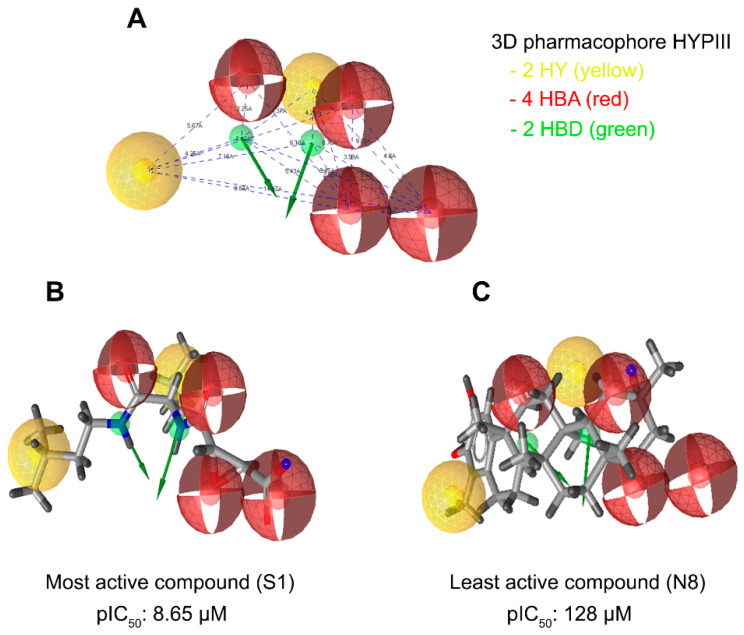
LigandScout was used to generate a three-dimensional pharmacophore model. (**A**) An eight-feature hypothesis (Hypo III) and its geometric constraints. Yellow indicates hydrophobic (HY), green indicates hydrogen bond donor (HBD), and red indicates hydrogen bond acceptor (HBA). (**B**) The best pharmacophore model (Hypo III) aligned to the training set’s most active molecule (compound S1; IC50 0.00224 µM) and (**C**) the inactive molecule compound N8 (IC50 125 µM). HY: hydrophobic, yellow; HBD: hydrogen bond donor, green; HBA: hydrogen bond acceptor, red.

The qualitative top 10 hypotheses were generated based on the training set molecules, which are tabulated in Table 2. The first three generated hypotheses were selected for the study because they had the highest pharmacophore-fit score in Hypo I (74.81, 7 matching features), Hypo II (75.24, 9 matching features), and Hypo III (75.98, 8 matching features). The matching features contained a combination of three chemical features, including hydrophobic (HY), hydrogen bond acceptor (HBA), and hydrogen bond donor (HBD). Hypothesis I (Hypo I) consisted of seven chemical features including one HY, five HBAs, and one HBD. A nine-feature hypothesis, Hypo II consisted of two HYs, five HBAs, and two HBDs. Hypo III contained two HYs, four HBAs, and two HBDs, constituting eight features overall (Figure 5A).

The pharmacophore fit score calculates the matching of number of pharmacophore features. This could predict the activity of compounds in the training set with the lowest deviation, while the RMSD represented pharmacophore alignment to estimate average activity, since the pharmacophore fit scores of the first three hypotheses were selected to validate the pharmacophore models. The validity of any pharmacophore model needs to be determined by applying that model to the test set to find out how correctly the model predicts the activity and, most importantly, whether it can correctly differentiate between active and inactive molecules. The pharmacophore model hypothesis was validated by assessing the predictive ability of the pharmacophore on the test set database consisting of 37 known inhibitors of cathepsin B, along with a subset of the World of Molecular Bioactivity (WOMBAT) database consisting of 741 molecules, active against different proteins than those used in present study, considered here as inactive. This validation gives confidence to select the best pharmacophore from amongst the three generated pharmacophore hypotheses. The results for pharmacophore validation are summarized in Table 3. A number of parameters such as hit list (Ht), active percentage yield (% Y), percentage ratio of active molecules in the hit list (% A), enrichment factor (E), false negatives, false positives, and goodness of hit score (GH) are presented (Table 3). Using the pharmacophore hypothesis Hypo I, 93 false positives were found, but only 28 active molecules were picked among the 37 in the test set. On the other hand, Hypo II showed 30 active hits, with 7 and 30 false negatives and false positives, respectively. The pharmacophore hypothesis Hypo III (Figure 5A) showed minimal false positives and negatives, good enrichment factor and goodness of fit score, and was considered to be the best model for virtual screening among the three pharmacophore hypotheses. Overall, amongst the 45 hit molecules, 35 molecules were observed to be correct, thus showing only 10 false positives and 2 false negatives. In addition, Hypo I, Hypo II, and Hypo III showed enrichment factors (E) of 4.98, 10.78, and 16.74, and GH scores of 0.32, 0.56, and 0.81, respectively indicating that the quality of the pharmacophore models is within an acceptable range. These results indicate that Hypo III is accurate enough to discriminate the active inhibitors from inactive or low-activity compounds.

In addition to the pharmacophore validation, Hypo III was tested against the predictive power of the pharmacophore model to differentiate between the most, moderately, and least active compounds in the training datasets. The training set molecules were classified into three categories based on their activity values: highly active (IC50 ≤ 0.3 µM, +++), moderately active (0.3 µM > IC50 < 2.5 µM, ++), and inactive (IC50 ≥ 2.5 µM, +). The error value is the ratio between the estimated and experimental activities. The positive error value indicates that the estimated IC50 value is higher than the experimental activity, whereas the negative error value indicates that the estimated IC50 value is much lower than the experimental activity. An error value of <10 signifies the prediction of activity lesser than one order of magnitude. Among the 24 training set compounds, only 2 compounds had an error value of greater than 3. The estimated activity values of the training set compounds were predicted with the same activity scale as the experimental activity, and are represented in Table 4. Among the 24 training set compounds, 1 active compound (+++) in the training set was estimated as moderately active (++), 1 moderately active compound (++) was estimated as active (+++), and 2 moderately active compounds (++) were estimated as inactive compounds (+). The remaining compounds were estimated in their activity scale by Hypo III (Table 4). The most active compounds of the training set (Compound S1, IC50: 8.65 µM) and their features flexibly aligned in Hypo III, and the least active compound (Compound N8, IC50: 125 µM) demonstrated poor alignment in the pharmacophore model (Figure 5B,C). The validated pharmacophore model could be used for searching structurally diverse compounds from the Maybridge molecular library database 

### 3.3. Virtual Screening and Hit Filtration

#### 3.3.1. Maybridge Database Screen and Fast Docking of Maybridge Molecular Library Hits Using AutoDock-Vina

**Figure 6:**
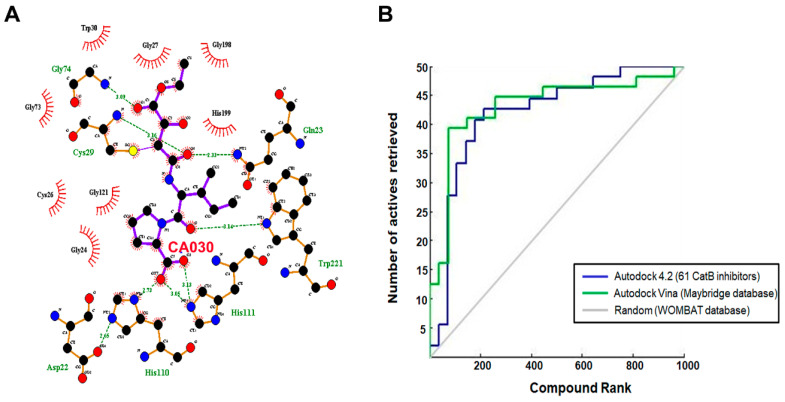
(**A**) Cathepsin B and ligand CA030 interaction determined through LigPlot. (**B**) Virtual screen ranking of the Maybridge compound library. A total of 1728 screened Maybridge compounds were docked to cathepsin B using AutoDock-Vina, then ranked by predicted binding energy. The plot shows the number of active compounds retrieved versus the total number selected (blue line for AutoDock 4.2 and green line for AutoDock-Vina). The grey line indicates the number of active molecules that would be expected to be returned based on a random selection of compounds.

The three-dimensional pharmacophore-validated Hypo III model was utilized for virtual screening of the diverse compounds in the Maybridge molecule library. A total of 60,538 small molecules from the Maybridge database were added in the LigandScout database library to identify potential hit molecules against the cathepsin B protein. As a result, 1728 compounds (2.9% of the Maybridge database) were perfectly matched and aligned to all features in the Hypo III model. These molecules were subjected to fast docking using ADt-Vina. From the resulting data, the compounds were ranked based on their predicted binding energies. These rankings were used to evaluate the ability of ADt-Vina to preferentially select the active compounds. The binding pocket of the cathepsin B protein (PDB: 1CSB) was considered based on the bound ligand (CA030) in the crystal structure (Figure 6A). In general, the default parameters were used for ADt-Vina. The docking program reported multiple conformations and associated binding energies. In case of ADt-Vina, the lowest energy conformation was selected. The compound rankings were determined and then compared against the active CA030 ligand. ADt-Vina reduced the Maybridge datasets to 176 molecule hits (0.3% of the Maybridge database) after applying a cutoff value of ≥−6.0 kcal/mol (Figure 3). As shown in Figure 6B, ADt-Vina displayed an accurate ranking of active compounds in cathepsin B. Quantified by an AUC measure (Table 5), ADt-Vina showed similar docking results for the Maybridge datasets (0.73) when compared to the AutoDock v4.2 (0.63) results. In terms of early recognition, utilizing the BEDROC measure, Vina (0.17) performed significantly better than AutoDock v4.2 (0.10) in the Maybridge database and the cathepsin B dataset (0.18 vs. 0.13) (Table 5). These molecules were further examined using Lipinski’s rule of five and visual inspection [65]. 

This led to a reduction in the number of virtual screening hits to 18 molecules. These 18 small hit molecules were subjected to re-docking analysis using AutoDock v4.2. The docking results of the 18 virtually screened small hit molecules are tabulated in Table 6, and their binding pose in the active site showed ligand conformations (Video S1).

#### 3.3.2. Structure Interaction Fingerprint Based Clustering

**Figure 7:**
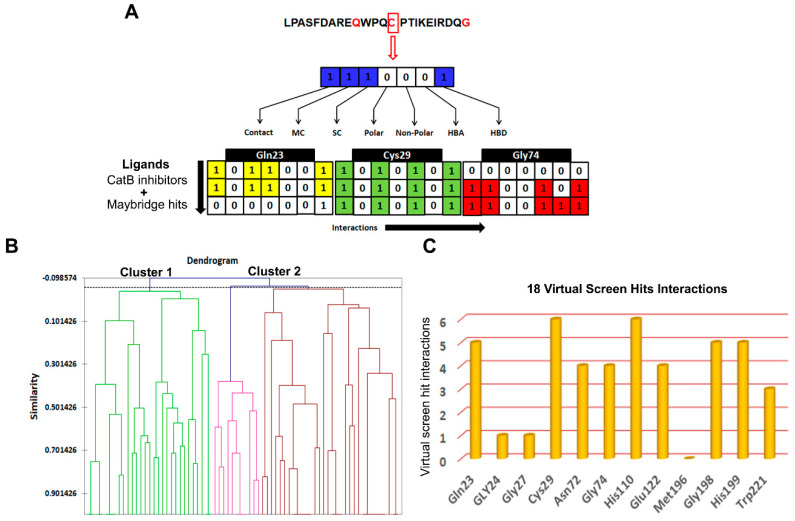
Screening based on structural interaction fingerprints (SIFts). (**A**) An illustrative representing the SIFt methodology. Fingerprints generated based on binding modes or pairwise interactions (H-bonds and vdW) formed between the proposed docking ligand conformation and a receptor. Step 1: identify the key binding residues of the receptor protein in the complex; step 2: represent each key residue by a bit string (contact, main chain (MC), side chain (SC), polar, non-polar, hydrogen bond acceptor (HBA), and hydrogen bond donor (HBD)) according to the kind of interaction at that residue; step 3: concatenate 7-bit strings of all key residues to form a unique fingerprint, called an SIFt. (**B**) Dendrogram derived from agglomerative hierarchical clustering of SIFts of known cathepsin B inhibitors and virtual screening hits. Tanimoto similarity coefficient was used to calculate the similarity between the SIFts. (**C**) The key residues of the cathepsin B protein involved in interaction with virtual screening hit molecules.

The SIFts were generated for 61 inhibitors of cathepsin B and 18 virtually screened hit molecules obtained after docking and scoring as described above (Figure 7A). The dendrogram was created by clustering SIFts of cathepsin B inhibitors and virtual screening of hit molecules and is shown in Figure 7B. The dendrogram showed two major clusters, each of which represents a distinct binding pattern in the ligand–protein complexes (Figure 7B). Cluster 1 (green color) was composed of 32.88% inhibitor molecules of cathepsin B (mostly less active) and 89.27% virtual screening hit molecules interacting with the cathepsin B protein. Similarly, Cluster 2 (brown and pink color) was composed of 67.12% cathepsin B inhibitors, which were mostly highly and moderately active, and 10.73% virtual screening hit molecules. Interestingly, each of these clusters was comprised of poses with similar binding patterns to the receptor; Cluster 1 contained molecules that represented distinct binding patterns that resulted in dissimilar interactions within the active site pocket formed by Gln23, His199, and Trp221 amino acid residues. Cluster 2 molecules bound in a similar manner to the known inhibitor in the X-ray crystal structures of PDB ID: 1CSB (Figure 7C). Most of the inhibitors of cathepsin B were located at the interface between the active molecule and the substrate-binding cleft (Supplementary Figure S1A). The moieties such as pyridine, sulfanyl and phenyl present in the hits belonging to Cluster 2 were predicted to occupy the protein’s active cleft. These played a critical role in peptide bond cleavage by Cys29 and interacted with His199 (Supplementary Figure S1B). These hits were buried deep within the binding groove and reached the catalytic residue Cys29 and were involved in π–π interactions. These moieties are also predicted to be involved in strong hydrogen bonding interactions with the conserved residues Gln23, Gly27, and Gly74. In most cases, the virtual screening hit molecules protrude outside of the catalytic pocket and occupy the neighborhood position composed of Gln23, Gly74, and Gly198 amino acid residues, belonging to the less active Cluster 1 (Supplementary Figure S1C).

Here, these hit molecules were involved in hydrophobic interactions with Tyr75 and Tyr177 residues (not shown here) and hydrogen bond interactions with residues His110 and His199. Some other interactions were also found to occur in most of the inhibitors of cathepsin B viz. a hydrogen bond between O3 of the pyridine base and the Trp221 side chain, a hydrogen bond between Glu122 and N1 of the pyridine ring, and O4 hydrogen bonds with the imidazole ring of His199.

### 3.4. Prioritization and Binding Modes of Hit Molecules

**Figure 8:**
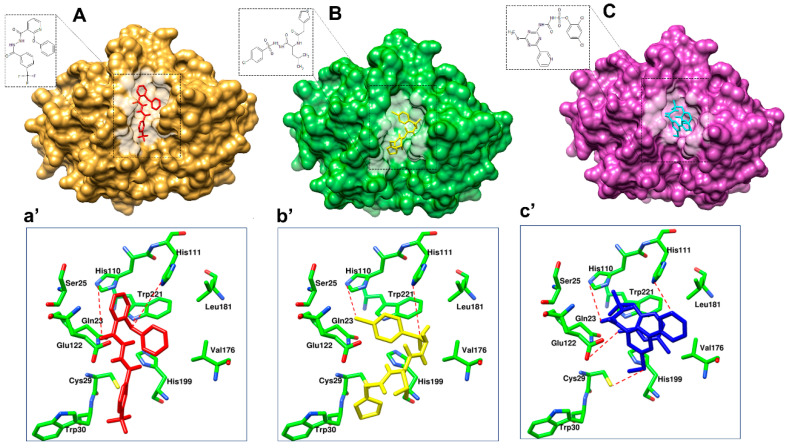
Binding modes of lead hits obtained after virtual screening. (**A**) BTB03075 ligand in red, cathepsin B protein surface view (golden yellow); (**a’**) Maybridge BTB03075 ligand demonstrating interaction with cathepsin B protein key amino acid residues. (**B**) KM02922 ligand in yellow, cathepsin B protein surface view (green); (**b’**) Maybridge KM02922 ligand demonstrating interaction with cathepsin B protein key amino acid residues. (**C**) RF02795 ligand in blue, cathepsin B protein surface view (purple); (**c’**) Maybridge RF02795 ligand demonstrating interaction with cathepsin B protein key amino acid residues.

In order to prioritize virtual screening hits, visual inspection of the clusters and their binding modes was carried out with the following considerations: (1) the compound should be from Cluster 2; (2) the degree of occupancy of the enzyme, in particular related to the achieved protein–ligand surface complementarities; (3) the distance and the orientation of the aromatic, aliphatic, or hydrophobic group in relation to His199 for π-stacking interaction; (4) the formation of a hydrogen bond with His110 and His111; and (5) the quality of the overall binding conformation. Overall, out of 18 compounds, 3 compounds—i.e., BTB03075, KM02922, and RF02795—showed acceptable binding poses and met all five of the mentioned criteria. Table 6 shows the binding scores of the 18 Maybridge hit compounds and, in comparison to the co-crystalized ligand (CA030), BTB03075, KM02922, and RF02795 demonstrated better binding scores. Figure 8 shows the binding pattern of the three representative compounds (BTB03075, KM02922, and RF02795) in the cathepsin B binding site (PDB ID: 1CSB). These molecules achieved hydrogen-bonding interactions with the key amino acids His110 and His111 in cathepsin B’s binding site via the imidazole side chain of the histidine amino acid. These require less distance to form hydrogen bonds with ligands, as the polar hydrogen atom of the imidazole ring acts as a hydrogen bond donor, while the basic nitrogen moiety is a hydrogen bond acceptor. The predicted binding modes of the Maybridge lead hits BTB03075, KM02922, and RF02795 are shown in Figure 8. Similarly, to most of the cathepsin B inhibitors, identified hits were all anchored to the cavity by hydrogen bonds between the ligand and Trp221, along with the p–p stacking interaction with His111 and His199. Aromatic rings of compounds BTB03075 (Figure 8A(a’)), KM02922, and RF02795 (Figure 8B(b’) and C(c’)) were embedded in the cavity formed by Trp221, Gln23, and His199 residues. Compound RF02795 showed the best results in terms of binding pattern and docking score amongst the 18 compounds in the 3 crystal structures, and its predicted binding affinity exceeded that of the co-crystalized ligands in the cathepsin B protein structure (Table 6). Regarding the binding pattern of compound RF02795, its triazinyl is well accommodated at the binding site, interacting with its amide functional group through hydrogen bonding with the key amino acids His110 and His111, fitting the hydrophobic methoxy phenyl ring in the vicinity of the hydrophobic side chains of the amino acids Trp30, Val176, Leu181, and Trp221, creating the hydrophobic pocket. These lead hits showed π–π stacking interactions and hydrogen bonding within the binding pocket of cathepsin B. The promising compounds all share common pharmacophoric features, such as a sulfur- and nitrogen-rich moiety (pyridine, sulfonyl, thiophenyl, triazinyl, etc.).

### 3.5. Molecular Dynamics Simulations

**Figure 9:**
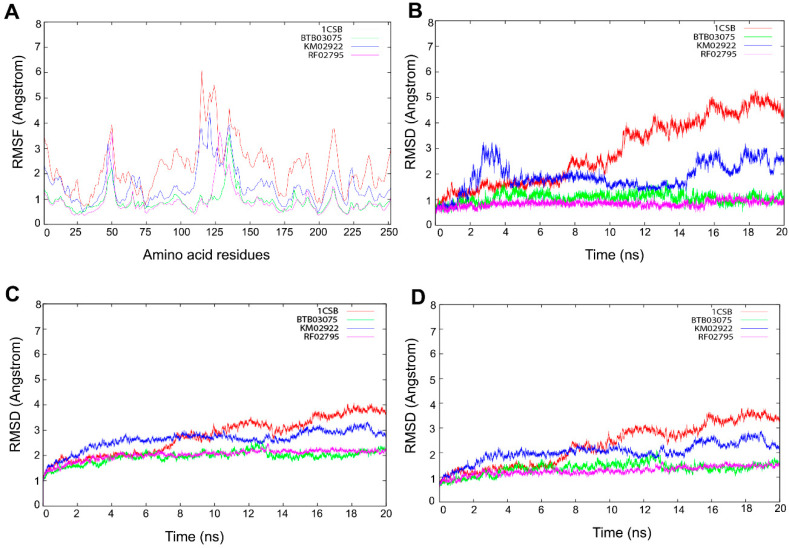
Molecular dynamic simulation performed for 20 ns. (**A**) The flexibility in the ligand–protein complex was examined by the root-mean-square fluctuation (RMSF) in each receptor–ligand complex. Evolution over time of the root-mean-square deviation (RMSD) of (**B**) binding site residues, (**C**) ligands, and (**D**) proteins.

BTB03075, KM02922, and RF02795 complexes with the cathepsin B protein were selected for molecular dynamics (MD) simulation to determine the long-term stability of the docked complex. An additional purpose underlying the MD studies was to investigate the positional and conformational changes of lead compounds in relation to the active pocket and specific residues, in order provide insight into the binding stability. The stability and interactions of the receptor–ligand complex was analyzed after 20-ns MD runs, and the root-mean-square deviations (RMSDs) of binding site residues and ligand trajectory were plotted against longer time frames (Figure 9). To elucidate the flexibility in the ligand–protein complex, we examined the root-mean-square fluctuation (RMSF) in each case of receptor–ligand complex (Figure 9A). RMSF analysis of protein–ligand complexes gave a maximum value of 6 Å for 1CSB-CA030. Initial stable RMSF flexibility was observed between 1 and 3 Å in the 1CSB protein backbone. Three large peaks of RMSF were seen between residues at 30–60, 100–130, and 200–225, arising from the α-helix, loop, and β-strand regions, and fluctuating between 3 and 6 Å (Figure 9A). This large peak was due to the binding of CA030, which affected the secondary structure of cathepsin B. There was no change in the secondary structure, although fluctuations in RMSF were observed in the α-helix, loop, and β-strand regions of cathepsin B upon BTB03075, KM02922, and RF02795 binding (Figure 9A). The average RMSD values of binding site residues of cathepsin B while bound with compounds BTB03075, KM02922, and RF02795 were observed to be 1.87, 0.92, and 0.91, respectively, suggesting stable binding sites. However, significant movements were observed for CA030, the bound inhibitor in the crystal structure of cathepsin B (PDB code 1CSB) (Figure 9B). MD further revealed that after 20 ns of simulations, no significant changes in ligand-docked conformation were observed for lead hits (BTB02922, KM02922, and RF02795) either, with average RMSD values of 2.93, 2.15, and 2.13 for BTB03075, KM02922, and RF02795, respectively (Figure 9C). Large movements were again observed in the case of the inhibitor-bound crystal structure of cathepsin B (Figure 9C,D). The results shown here confirm the docking stability of the predicted binding modes of BTB02922, KM02922, and RF02795. The trajectory generated from 20-ns MD simulation was then used to calculate ligand binding free energy using the MM-PBSA [66] and MM-GBSA methods [67]. Both the MM-PBSA and MM-GBSA methods can be used to quickly reproduce relative binding affinities for a set of ligands, with reasonable accuracy [68,69]. The MM-PBSA- and MM-GBSA-predicted ligand binding free energies are presented in Table 7, and strong correlation was observed between ligand binding free energies calculated from both the MM-PBSA and MM-GBSA methods. The MM-PBSA- and MM-GBSA-predicted ligand binding free energies for the three virtual screening hits were either much lower than or comparable with the known inhibitor of cathepsin B (Supplementary Figure S2).

### 3.6. Cathepsin B Inhibition Activity of Virtually Screened Hit Compounds

**Figure 10:**
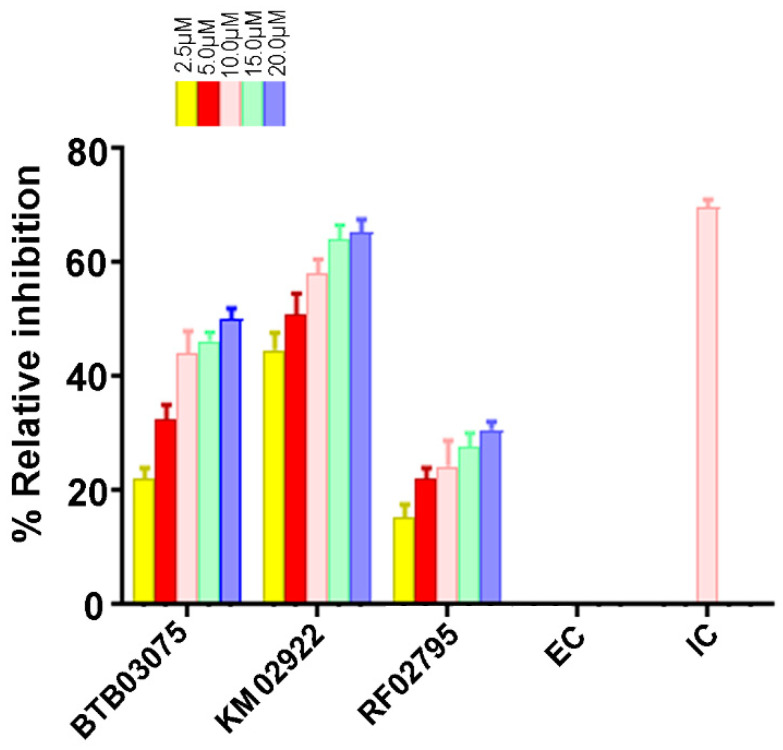
The activity of virtually screened molecules using a cathepsin B inhibitory screening assay kit. Dose-dependent inhibition of cathepsin B activity by the virtually screened molecules. F-F-FMK was used as a standard inhibitor of cathepsin B activity (positive control, 10 μM concentration), represented as inhibitor control (IC), whereas the solvent with enzyme only (negative controls) is represented as enzyme control (EC). Experiments were performed in triplicate. Data are represented as the mean ± SEM.

To validate our mixed-feature 3D pharmacophore modeling and ligand-based virtual screening approach, we tested the cathepsin B inhibitory effects of the test compounds in vitro. The three hit molecules BTB02922, KM02922, and RF02795 were selected after passing our high-throughput screening protocol and showing higher pose stability in MD studies. These drug-like compounds were incubated with human cathepsin B in a concentration-dependent manner. F-F-FMK was used as a positive control and was tested for % inhibition at a concentration of 10 µM only. The compounds—namely, BTB02922, KM02922, and RF02795—were tested at dose-dependent concentrations of 2.5 µM, 5.0 µM, 10.0 µM, 15.0 µM, and 20.0 µM, and exhibited inhibition of the cathepsin B enzyme in the kinetic assay. KM02922 showed the best relative inhibitory activity at a relative inhibition of 44.67, 51.0, and 58% at concentrations of 2.5, 5.0, and 10 µM, respectively (Figure 10). The second-best compound was BTB02922, which also showed a high percentage relative inhibition, but did not reach the level of compound KM02922. The control inhibitor (F-F-FMK) demonstrated 69.67% relative inhibition of cathepsin B at a concentration of 10 µM.

## 4. Discussion

AD is a neurodegenerative disease that affects neuronal cells, eventually leading to irreversible damage and death of neurons [70,71]. Identification of target-directed inhibitors to combat this complex disease can greatly help in mechanism-based drug development. Aβ plaques and tau hyperphosphorylation are two hallmark features of AD, and various reports have indicated that these events are not mutually exclusive [71,72,73]. The development of inhibitors against the cysteine protease cathepsin B, which participates in the breakdown of APP and is involved in Aβ pathology by acting as a beta-secretase, may be of vital importance. Hook et al. reported that the cysteine protease inhibitor E64d reduces Aβ in the brain and improves memory deficits in AD animal models by inhibiting cathepsin B, but not beta-secretase activity [74]. A neuroprotective role of cathepsin B is also reported in some of the studies by lowering Aβ levels and improving neuronal dysfunction in AD animal models [75,76,77]. Thus, a dual role of cathepsin B as a neurodegenerative and neuroprotective enzyme provides an important therapeutic target for AD. We have previously reported novel molecular scaffolds for Alzheimer’s acetylcholinesterase inhibitors using a rational drug design approach [42].

The current study investigated the step-by-step in silico drug screening protocol to identify new small molecules with cathepsin B inhibitory activity using a combination of molecular docking, pharmacophore mapping, SIFts, and molecular dynamics approaches. Firstly, anti-cathepsin-B compounds of both natural and synthetic origins were initially docked to the cathepsin B active site using AutoDock v4.2 (Table 1). The molecular docking technique is a fast method to analyze the binding affinity of the protein–ligand interactions and has been implicated in the discovery of several drug molecules [78,79]. A ligand-based virtual screening (LBVS) pharmacophore model was developed using the ligandset module in LigandScout 3.1 [45]. This method was useful to identify the structural features of compounds interacting with cathepsin B, and to define the three-dimensional alignment of unique pharmacophoric features [80]. The LBVS method exploits the information of compounds with inhibitory activity in order to retain important chemical and physical properties required for the ligand to bind to the protein and elicit biological activity and a pharmacological response [81]. Importantly, all LBVS is based on the common conception that all bioactive compounds interact with the protein (receptor) in a similar manner, leaving ample diversity in the dataset with similar functional groups, which can later be converted to pharmacophoric features that are related to ligand biological activity [82]. Therefore, these pharmacophoric features could underscore the chemical features of cathepsin B interaction. To generate 3D pharmacophore models, 24 potent cathepsin B inhibitors of both natural and synthetic origins were randomly selected as training set compounds, mainly comprising a broad range of small scaffold molecules previously reported as having anti-cathepsin-B activity (Figure 2, marked with *). The training set represents the following classes: ethylene oxide, benzopyran, nitramide, pyrrolidine, diazonium, triazine, and sulfonyl pyrazol (Figure 2, Table S1). Ten different hypotheses (Hypo I to Hypo X) were generated using pharmacophore-fit and atom-overlap scoring functions, as shown in Table 2. The refinement of the hypothesis was carried out by adding the WOMBAT database of 741 compounds inactive against cathepsin B. This refinement process allowed us to validate the three pharmacophore hypotheses with the highest goodness of fit scores and enrichment factors. Finally, Hypo III, with eight features (2 HYs, 4 HBAs, and 2 HBDs), was selected because it demonstrated the best features to distinguish between the active and inactive compound libraries described in Table 3. It was noted that all LBVS models share HBA and HBD features, reflecting that the group present in all molecules that acts as H-bond acceptors and donors—expected to form H-bond interactions at the cathepsin B catalytic site with Cys29 and His199 (Figure 4A)—is crucial for anti-cathepsin-B activity [10].

The greatest challenge of the pharmacophore model is to discriminate the active and inactive compounds and predict the activity of the test/training sets at the same time. Thus, in order to carefully examine the quality of the proposed pharmacophore models, the following validation methods were used: (1) formation of the raining set to confirm that the used ligands are detected; (2) differentiation of the highly active, moderately active, and less active compounds in the test set; and (3) non-active molecules (decoys) and active molecules to validate the predictability of the hypothesis, in order to pick the active from the unknown database. Furthermore, the performance of the 3D pharmacophore model is more accurately measured in larger, rather than smaller, datasets [83]; thus, the validation dataset consisted of 796 (759 decoys and 37 active) molecules. Hypo III correctly estimated the highly active (IC50 ≤ 0.3 µM, +++), moderately active (0.3 µM > IC50 < 2.5 µM, ++), and less active (IC50 ≥ 2.5 µM, +) molecules (Figure 5, Table 4).

After validation of pharmacophore Hypo III, it was employed for rapid screening of the drug-like chemical library obtained from the Maybridge database [84]. As a benchmark of the current study, small molecules were required in order to significantly reduce the database size by both satisfying pharmacophores and detecting the promising hits correctly. Around 1728 drug-like compounds matched to the Hypo III pharmacophore model, thereby yielding 2.85% of the Maybridge screen library. In addition, fast docking of 1728 drug-like molecules using ADt-Vena was employed to predict the binding conformation and classify the ability of these molecules to form a stable complex within the binding cavity of the cathepsin B protein [85]. These hits were also predicted online for blood–brain barrier (BBB) penetration using an online BBB predictor [86] (Figure 3), reduced to 176 drug-like compounds. Moreover, the analysis of the ROC curves also assesses the performance of the pharmacophore hypotheses in separating active from decoy compounds (Figure 6, Table 6), and could be effectively used to find novel anti-cathepsin-B compounds. To this end, the pharmacophore model Hypo III, was employed to rapidly screen 176 drug-like compounds passing through visual inspection, Lipinski’s rule of five, and docking score; a total of 18 hits survived [87,88]. The ligand-binding energies for the protein–ligand complexes of each one of these 18 hits docked to the cathepsin B protein, and were obtained by performing LGA calculation (Table 6) [89]. LGA estimates the binding affinity for flexible ligand–receptor docking, which allows the accommodation of many degrees of freedom. The top docked hits, with binding energy scores < −8.00 kcal mol^−1^, were sorted based on binding energy score (Table 6). The binding poses of these ligands were analyzed for their relative positions and orientations, and the various molecular interactions they formed in the binding site of cathepsin B, through the generation of SIFt profiles, which were utilized as a virtual screening tool to predict protein–ligand interaction contours when studied in the same target protein (Figure 7). Key representations of the interactions in binding pockets were similarly employed by Ritschel et al. for quantifying the similarities of binding site subpockets in order to explain the pharmacological effects of statin (HMG-CoA reductase) inhibitors based on pharmacophore fingerprints [90]. The best docking poses from known cathepsin B inhibitors and virtual screening hits were clustered using similarity matrices (Jaccard–Tanimoto coefficient) between all of the fingerprints (Figure 7B,C). The dataset used for the generation of SIFt profiles consisted of 79 molecules (61 known cathepsin B inhibitors and 18 virtual screening hits). Figure 7B shows a dendrogram of the less active (Cluster 1) and most active (Cluster 2) clusters obtained via the hierarchical clustering approach [53]. The binding mode of hit molecules presented in Cluster 2, identified through hierarchical clustering, suggested that the bound conformation of virtual screening hits strongly mimics the observed conformation of cathepsin B inhibitors at the protein-binding site. In the specialized case environment, the thiol and imidazole side chains of cathepsin B Cys29 and His199 amino acid residues form an ion pair [91]. The cleavage is then mediated by nucleophilic attack by S- from Cys29 on the carbon atom, followed by proton donation from His199 [91]. The SIFt outcome was promising because, although the SIFt dataset was assigned to the less active side of the cathepsin B inhibitors, ultimately, smaller clusters of active compounds containing lead-like molecules were obtained. These molecules form hydrogen bonds with His110 and His111 and π-stacking interactions with His199 (Figure 8). The promising lead compounds (BTB03075, KM02922, and RF02795) share common pharmacophoric features, such as a sulfur- and nitrogen-rich moiety (pyridine, sulfonyl, thiophenyl, triazinyl, etc.).

Molecular dynamic simulations are a key component of the drug discovery process to assess the conformational stability of the docked protein–ligand complexes, as well as to analyze the molecular interactions between the ligands and the protein-binding site residues [92,93]. The top three prioritized drug-like molecules, along with the cathepsin B crystal structure (PDB 1D: 1CSB) used for the virtual screening, were subjected to the same simulation parameters (Supplementary Materials). The RMSF and RMSD analyses revealed that, for all four systems, there was minimal fluctuation in the flexibility of the protein backbone atoms, suggesting that binding of all three ligands remained stable, and that ligand binding did not affect protein stability throughout the course of the simulation run (Figure 9). In rational drug design, MM-PBSA and MM-GBSA are powerful tools for optimizing lead compounds, because they can critically analyze the interactions of ligand–receptor bonds [55]. It has been suggested that electrostatic interactions dominate over non-covalent bonding in the recognition of molecular interactions between drug and target molecules [94]. However, this cannot always be true, as the geometric shape is also an important aspect, which is affected by other forces, such as van der Waals interactions, solvation/desolvation energy, and entropy. MM-PBSA and MM-GBSA predicted the binding free energy components of CA030, BTB03075, KM02922, and RF02795, as shown in Table 7 and Supplementary Figure S2. There are numerous studies where the critical interactions of the ligand–receptor pairs employed MM-PBSA and MM-GBSA approaches in a computing framework [95,96,97,98]. For example, by using biological and computational MM-PBSA free energy evaluation, a series of resveratrol derivatives to provide an explanation for the anti-β-secretase (BACE-1 in neurons involved in Aβ deposition in the brain) activity in BACE-1 and oxytosis inhibition assay [99], while in another study, application of MD and explicit water thermodynamics was used to identify a new class of cathepsin B inhibitors [100].

## 5. Conclusions

In this study, we carried out LBVS of 60,538 Maybridge drug-like molecules by screening these compounds through a 3D pharmacophore model generated from novel inhibitors of cathepsin B, of both natural and synthetic origins. The pharmacophore model Hypo III represents a spatial orientation of eight specific molecular features, including two HYs, four HBAs, and two HBDs, which facilitate sufficient interactions for binding to cathepsin B and, consequently, the potential inhibition of its protease function. The most active molecule in the training set fits the pharmacophore model perfectly with the highest scores. We found 1728 compounds with the relevant eight-feature pharmacophore. These 1728 Maybridge compounds were then tested by fast docking using AutoDock-Vina to perform virtual screening and assess their ability to cross the BBB, which reduced the number of drug-like compounds to 176. Docking score and Lipinski’s rule of five further lowered the number to 18 compounds. SIFt mapping made it possible to quickly shortlist promising compounds and automate the visualization of putative active binding modes using hierarchical clustering. The active Cluster 2 demonstrates important interactions, including hydrogen bonding and π–π stacking with the key residues in the cathepsin B binding site residue, viz. Gln23, Gly24, Gly27, Asn72, Gly74, His110, Glu122, Met196, Gly198, His199, and Trp221. Enzyme assay was performed on three virtually screened compounds to validate the pharmacophore hypothesis, and KM02922 was found to be a potent cathepsin B inhibitor. However, compounds BTB03075 and RF02795 showed a lower percentage of relative inhibitory activity. Furthermore, the stability of the lead molecules was subjected to 20-ns scale MD simulations in order to satisfy the selected pharmacophore model. MM-PBSA and MM-GBSA calculation are predicted to inhibit the cathepsin B activity, with significant binding energies. We successfully identified new entities—including pyridine, acetamide, and benzohydrazide derivatives—that have not been previously characterized in the published literature as AD cathepsin B inhibitors. These findings may be useful from a medicinal chemistry and combinatorial chemistry perspective to obtain new molecular starting points for the design and optimization of novel cathepsin B inhibitors for AD.

## Figures and Tables

**Table 1:** Dataset of 61 Cathepsin B inhibitors and reference ligand (CA030) with corresponding energies obtained from the docking test performed using AutoDock v4.2 program. The training sets are denoted with “*”.

S.No	C.Name	*BE^e^* (kcal/mol)	*Ki* (µM)	*IME^e^* (kcal/mol)	V_dw_-H_b_-D_s_ (kcal/mol)	*E^e^* (kcal/mol)	*IE^e^* (kcal/mol)	*TFE^e^* (kcal/mol)
1	CA030	−5.89	48.10	−6.32	−6.77	+0.44	+0.16	+0.27
	N1 *	−4.11	964.9	−6.45	−7.10	+0.66	−1.23	+3.57
2	N2 *	−7.30	4.47	−8.96	−8.18	−0.78	−2.45	+4.12
3	N3	−8.15	1.06	−8.49	−8.49	+0.00	−2.13	+2.47
4	N4 *	−10.28	29.17	−9.54	−9.31	−0.22	−2.94	+2.20
5	N5	−5.37	115.2	−8.85	−7.81	−1.05	−2.01	+5.49
6	N6 *	−6.55	15.77	−6.75	−4.98	−1.77	−3.09	+3.29
7	N7	−6.07	35.48	−6.00	−5.95	−0.05	−1.72	+1.65
8	N8 *	−7.11	6.17	−8.15	−8.96	+0.81	+0.22	+0.82
9	N9	−6.61	14.25	−8.28	−7.61	−0.67	−2.72	+4.39
10	N10 *	−5.71	65.31	−5.76	−5.65	−0.11	−0.77	+0.82
11	N11	−10.35	26.10	−9.91	−10.00	+0.08	−1.53	+1.10
12	N12	−6.29	24.31	−6.29	−6.34	+0.04	+0.00	+0.00
13	N13 *	−6.26	25.83	−8.34	−7.26	−1.08	−1.21	+3.29
14	N14 *	−9.47	97.11	−7.27	−7.76	+0.49	−0.95	+2.74
15	N15 *	−6.20	28.60	−7.08	−6.94	−0.15	−2.41	+3.29
16	N16	−5.30	130.1	−7.19	−6.13	−1.05	−1.68	+3.57
17	N17 *	−8.57	522.5	−8.31	−8.23	−0.08	−2.46	+2.20
18	N18	−7.16	5.61	−8.16	−6.46	−6.46	−0.10	+1.10
19	N19	−5.93	45.25	−7.83	−7.67	−0.15	−1.94	+3.84
20	N20	−5.92	45.44	−7.52	−7.45	−0.07	−1.42	+3.02
21	N21 *	−9.35	0.134	−9.59	−9.40	−0.19	−2.24	+2.47
22	N22	−5.03	205.2	−5.03	−4.95	−0.08	+0.00	+0.00
23	S1 *	−5.70	66.00	−7.28	−7.52	+0.23	−1.16	+2.74
24	S2 *	−6.42	19.52	−8.01	−8.01	+0.16	−1.16	+2.74
25	S3	−6.65	13.30	−8.07	−6.52	−1.55	−2.43	+3.84
26	S4 *	−6.38	21.20	−8.16	−8.19	+0.03	−0.96	+2.74
27	S5	−8.05	1.26	−6.94	−6.94	−0.08	−2.20	+1.10
28	S6	−5.55	84.91	−6.15	−6.02	−0.13	−0.51	+1.10
29	S7 *	−6.26	25.76	−8.32	−8.30	−0.03	+0.42	+1.65
30	S8	−7.56	2.89	−8.65	−8.50	−0.15	−0.83	+1.92
31	S9 *	−6.86	9.35	−7.11	−7.11	−0.06	−0.30	−0.30
32	S10	−7.92	1.56	−8.14	−8.10	−8.10	−1.42	+1.65
33	S11	−7.71	2.23	−7.80	−7.77	−0.04	−1.83	+1.92
34	S12	−6.63	13.86	−6.46	−6.33	−0.14	−1.81	+1.65
35	S13	−5.61	77.13	−5.72	−5.62	−0.10	−0.44	+0.55
36	S14	−5.84	52.54	−6.80	−5.14	−1.66	−0.41	+1.37
37	S15	−7.21	5.18	−8.10	−7.88	−0.22	−1.03	+1.92
38	S16	−5.15	16.76	−5.39	−5.28	−0.11	−0.31	+0.55
39	S17 *	−9.34	0.14	−9.77	−9.55	−0.23	−1.48	+1.92
40	S18 *	−7.18	5.50	−7.85	−7.82	−0.04	−0.42	+1.10
41	S19 *	−8.07	1.21	−9.05	−6.28	−2.77	−1.22	+2.20
42	S20	−7.19	5.36	−8.17	−7.99	−0.18	−0.67	+1.65
43	S21	−6.95	8.05	−7.99	−8.02	+0.03	−0.88	+1.92
44	S22 *	−7.41	3.72	−8.12	−8.18	+0.06	−0.66	+1.37
45	S23	−6.43	19.44	−7.98	−7.95	−0.03	−0.92	+2.47
46	S24	−6.98	7.70	−7.66	−4.46	−3.20	−0.14	+0.82
47	S25 *	−7.20	5.27	−7.01	−6.92	−0.09	−0.74	+0.55
48	S26	−7.86	1.73	−8.21	−8.08	−0.13	−1.02	+1.37
49	S27 *	−7.97	1.43	−8.72	−8.69	−0.03	−0.63	+1.37
50	S28	−6.99	7.52	−7.17	−7.21	+0.04	−0.64	+0.82
51	S29	−6.91	8.67	−7.23	−7.14	−0.10	−0.50	+0.82
52	S30	−6.16	30.57	−5.97	−5.86	−0.11	−0.74	+0.55
53	S31	−6.70	12.23	−7.66	−7.50	−0.16	−0.96	+1.92
54	S32 *	−6.59	14.68	−8.48	−8.28	−0.21	+0.24	+1.65
55	S33	−5.19	155.8	−6.13	−6.05	−0.08	+0.11	+0.82
56	S34 *	−7.10	6.20	−7.82	−7.78	−0.04	−0.93	+1.65
57	S35	−7.25	4.83	−7.85	−7.69	−0.16	−1.05	+1.65
58	S36	−9.37	135.9	−10.31	−10.15	−0.16	−1.52	+2.47
59	S37	−7.19	5.40	−8.38	−8.39	+0.02	−0.45	+1.65
60	S38	−6.23	27.29	−7.21	−7.13	−0.08	−1.76	+2.74
61	S39	−6.82	10.08	−6.65	−6.63	−0.02	−1.26	+1.10

*BE^e^* Estimated binding free energy in kcal mol^−1^; *Ki* Inhibitory constant in micro-molar; *IME^e^* Final Intermolecular Energy in kcal mol^−1^; V_dw_–H_b_–D_s_ Van der waals-hydrogen bond-desolvation energy component of binding free energy in kcal mol^−1^; *E^e^* Electrostatic energy in kcal mol^−1^; *IE^e^* Final total internal energy in kcal mol^−1^; *TFE^e^* Torsional free energy in kcal mol^−1^.

**Table 2:** Details of top ten hypotheses generated using pharmcophore-fit and atom overlap scoring function.

Hypothesis Name	Features ^a^	Matching Features	Pharmacohore-Fit Score ^b^
Hyp I	HY, HBA, HBA, HBA, HBA, HBA, HBD	7	74.81
Hyp II	HY, HY, HBA, HBA, HBA, HBA, HBA, HBD, HBD	9	75.24
Hyp III	HY, HY, HBA, HBA, HBA, HBA, HBD, HBD	8	75.98
Hyp IV	HY, HBA, HBA, HBD, HBD, HBD	6	74.22
Hyp V	HY, HY, HBA, HBA, HBA, HBA	6	74.17
Hyp VI	HY, HBA, HBA, HBA, HBD, HBD	6	73.96
Hyp VII	HY, HY, HBA, HBA, HBA, HBA, HBA, HBD	9	74.65
Hyp VIII	HY, HBA, HBA, HBA, HBD, HBD, HBD	7	74.57
Hyp IX	HY, HY, HBA, HBA, HBD, HBD, HBD	7	73.42
Hyp X	HY, HY, HBA, HBA, HBA, HBA, HBA, HBD	8	74.11

^a^ HY = Hydrophobic; HBA = Hydrogen Bond Acceptor; HBD = Hydrogen Bond Donor. ^b^ Higher the pharmacophore-fit score, lesser the probability of chance correlation. The best hypothesis shows the highest value.

**Table 3:** Statistical parameters employed in mixed feature ligand-based pharmacophore generation of cathepsin B test set molecules.

No.	Parameter	Hypo I	Hypo II	Hypo III
1	Total number of molecules in the database (D)	796	796	796
2	Total number of actives in the database (A)	37	37	37
3	Total hits (H_t_)	121	60	45
4	Active hits (H_a_)	28	30	35
5	% Yield of actives (H_a_/H_t_ × 100)	23.15	50	77.78
6	% Ratio of actives (H_a_/A × 100)	75.68	81.1	94.6
7	Enrichment factor (E) ^a^	4.98	10.78	16.74
8	False negatives (A − H_a_)	9	7	2
9	False positives (H_t_ − H_a_)	93	30	10
10	Goodness of hit score (GH) ^b^	0.32	0.56	0.81

^a^ E = H_a_ × D/H_t_ × A. ^b^ GH = (Ha/4 Ht × A) (3A + Ht) × (1 − [(Ht − Ha)/(D − A)].

**Table 4:** Experimental and estimated IC_50_ values of the training (shown *) and test set compounds based on the pharmacophore hypothesis ‘Hyp III’.

Compound	pIC_50_		Error ^a^	Fit Value ^b^	Activity Scale ^c^	
	Experimental	Estimated			Experimental	Estimated
* N1	7.14	6.22	−1.1	74.21	+++	+++
* N2	6.89	7.24	+2.3	73.29	+++	++
N3	5.76	5.45	+3.2	72.34	++	++
* N4	6.21	6.33	+0.9	71.91	++	++
N5	6.23	6.1	+2.9	70.14	++	+
* N6	6.58	6.44	−1.3	72.14	+++	+++
N7	6.55	6.44	−1.8	71.36	+++	+++
* N8	3.91	4.23	+2.4	69.15	+	+
N9	5.59	6.27	−1.9	71.85	+	+
* N10	8.03	8.11	+1.2	75.12	+++	+++
N11	9.1	9.22	+2.5	69.71	+++	++
N12	7.99	8.14	+1.5	72.49	+++	+++
* N13	8.28	8.22	−0.6	75.16	+++	+++
* N14	8.47	8.84	+2.2	76.12	+++	+++
* N15	6.57	8.53	+3.2	73.11	+++	+++
N16	6.49	6.78	−1.5	72.44	++	++
* N17	6.26	8.23	+3.1	71.94	++	+++
N18	6.24	6.21	+1.3	72.45	++	++
N19	6.36	6.26	+2.2	71.88	++	++
N20	8.47	7.98	+3.7	72.67	+++	++
* N21	5.77	5.72	−0.5	71.26	++	++
N22	7.34	7.31	+2.8	72..12	+++	+++
* S1	8.65	8.62	−0.3	77.68	+++	+++
* S2	8.55	8.53	−0.4	76.23	+++	+++
S3	8.4	8.34	−0.5	70.11	+++	+++
* S4	8.03	8.01	−0.3	75.1	+++	+++
S5	4.47	3.29	+3.1	72.4	+	+
S6	4.94	5.13	−1.6	71.85	+	+
* S7	5.7	4.91	−1.8	71.22	++	+
S8	4.91	4.9	+0.4	69.36	+	+
* S9	7.15	7.78	+1.6	74.29	+++	+++
S10	5.9	5.56	+3.4	71.36	++	+
S11	5.2	5.16	−0.3	70.88	+	+
S12	5.76	5.73	+2.4	71.73	++	++
S13	7.14	6.78	+1.2	71.49	+++	++
S14	4.35	4.36	−0.1	70.34	+	+
S15	6.03	6.05	+1.9	71.13	++	++
S16	5.67	5.41	+3.7	72.55	++	+
* S17	5.02	6.01	+1.4	70.63	+	+
* S18	5.17	5.23	+1.1	70.89	+	+
* S19	4.71	4.67	−1.6	70.25	+	+
S20	5.05	5.03	+0.4	71.61	+	+
S21	4.39	4.38	+0.6	70.78	+	+
* S22	4.41	4.41	−0.8	70.16	+	+
S23	5.68	5.7	+1.5	71.64	++	++
S24	4.85	4.83	+2.1	69.52	+	+
* S25	5.38	5.96	+1.7	71.13	+	+
S26	4.43	4.42	+1.4	71.47	+	+
* S27	4.34	4.39	+0.6	69.67	+	+
S28	5.15	5.17	−0.5	71.45	+	+
S29	5.49	5.48	+1.6	70.43	+	+
S30	5.93	5.91	+1.1	69.71	++	++
S31	6.16	6.15	+1.1	72.43	++	++
* S32	6.36	7.63	+1.9	71.98	++	+
S33	6.07	6.08	+1.3	69.11	++	++
* S34	6.61	5.56	−1.8	73.45	+++	+++
S35	5.07	5.06	−1.1	74.23	+	+
S36	4.74	4.75	+1.2	71.26	+	+
S37	5.65	5.41	+1.3	72.84	++	+
S38	6.92	6.94	+1.1	71.04	+++	+++
S39	6.09	6.07	−0.3	70.09	++	++

^a^ Positive value indicates that the estimated IC_50_ is higher than the experimental IC_50_; negative value indicates that the estimated IC_50_ is lower than the experimental IC_50_. ^b^ Fit value indicates how well the features in the pharmacophore map the chemical features in the compound. ^c^ Activity scale: active, +++, IC_50_ ≤ 0.3 µM; moderate active, ++, 0.3 µM > IC_50_ < 2.5 µM; less active, +, IC_50_ ≥ 2.5 µM).

**Table 5:** Virtual screen statistics. Area under the curve (AUC) and Boltzmann-enhanced discrimination of receiver operating characteristic (BEDROC) 20 values were calculated based on the data shown in Figure 6. *p*-values were estimated using a bootstrap procedure based on 100,000 random rankings of the active compounds.

Library		AUC	*p*-Value	BEDROC 20	*p*-Value
Cathepsin B dataset	Autodock v4.2 Adt-Vina	0.74	0.00039	0.13	0.12
		0.72	0.00086	0.18	0.03
Maybridge dataset	Autodock v4.2 ADt-Vina	0.63	0.00067	0.10	0.51
		0.73	0.00088	0.17	0.0075

**Table 6:** Screened 18 hits from Maybridge database and corresponding energies values obtained from the re-docking validation test performed using AutoDock program.

S.No	C.Name	*BE^e^* (kcal/mol)	*Ki* (µM)	*IME^e^* (kcal/mol)	V_dw_-H_b_-D_s_ (kcal/mol)	*E^e^* (kcal/mol)	*IE^e^* (kcal/mol)	*TFE^e^* (kcal/mol)
1	AW01196	−6.52	22.02	−6.37	−6.83	+0.46	−0.62	+2.47
2	BTB03075	−8.17	1.02	−8.45	−8.42	−0.03	−1.37	+1.65
3	BTB11814	−7.07	6.52	−8.04	−7.94	−0.10	−1.50	+2.47
4	HTS05162	−7.65	2.46	−7.88	−7.77	−0.11	−0.87	+1.10
5	JFD02054	−6.08	35.21	−7.56	−7.60	+0.04	−1.26	+2.74
6	JP00474	−7.60	2.69	−8.08	−8.45	+0.36	−1.71	+2.20
7	KM02757	−7.31	4.41	−7.72	−7.43	−0.28	−2.61	+3.02
8	KM02759	−8.16	1.04	−9.25	−9.19	−0.06	−1.93	+3.02
9	KM02760	−7.31	4.41	−7.42	−7.37	−0.05	−2.91	+3.02
10	KM02777	−7.53	3.01	−8.22	−8.25	+0.03	−2.61	+3.29
11	KM02922	−8.19	0.99	−8.47	−8.47	−0.03	−2.19	+2.47
12	RF02795	−9.18	0.18	−9.44	−8.00	−1.44	−0.84	+1.10
13	RJC00586	−6.29	24.43	−7.84	−7.81	−0.03	−1.20	+2.74
14	S12294	−6.87	20.92	−6.45	−6.75	+0.30	−0.79	+1.37
15	SPB02418	−7.15	5.75	−7.91	−7.62	−0.29	−0.61	+1.37
16	SPB03394	−6.76	11.11	−7.01	−7.08	+0.08	−0.85	+1.10
17	SPB03600	−8.01	1.35	−8.27	−8.15	−0.12	−0.83	+1.10
18	SPB08054	−6.56	15.53	−8.21	−8.16	−0.05	−0.82	+2.47

*BE^e^* Estimated binding free energy in kcal mol^−1^; *Ki* Inhibitory constant in micro-molar; *IME^e^* Final Intermolecular Energy in kcal mol^−1^; V_dw_–H_b_–D_s_ Van der waals-hydrogen bond-desolvation energy component of binding free energy in kcal mol^−1^; *E^e^* Electrostatic energy in kcal mol^−1^; *IE^e^* Final total internal energy in kcal mol^−1^; *TFE^e^* Torsional free energy in kcal mol^−1^.

**Table 7:** MM-PBSA and MM-GBSA predicted binding free energy components and standard deviations for the Maybridge virtual screen hits and reference ligand CA030.

Compounds	Δ*E*_vdw_	Δ*E*_elec_	Δ*G*_MM_	Δ*G^PB^*_polar_ _solvation_	Δ*G^PB^*_nonpolar solvation_	Δ*G^GB^*_polar solvation_	Δ*G^GB^*_nonpolar solvation_	Δ*G^MM−PBSA binding^*	Δ*G^MM−GBSA binding^*
CA030	−25.81	−40.86	−66.67	41.45	−5.42	36.62	−5.42	−25.22	−30.06
BTB03075	−19.43	−39.35	−58.78	39.53	−5.94	31.97	−5.64	−19.25	−26.81
KM02922	−29.50	−51.66	−81.66	46.48	−6.61	38.96	−6.61	−34.68	−42.20
RF02795	−17.39	−58.86	−76.25	44.32	−6.38	37.24	−6.38	−31.93	−39.01

## Supplementary Materials

The following are available online at https://www.mdpi.com/article/10.3390/cells10081946/s1, Figure S1: (A) Active site of cathepsin B, displaying the binding mode of some cathepsin B inhibitors; (B) virtual screening hits belonging to active Cluster 2 compounds displaying π–π interactions; and (C) virtual screening hits belonging to less active Cluster 1, which contains molecules with distinct binding modes. Some representative images of structures are shown; Figure S2: Correlation plot between MM-PBSA- and MM-GBSA-predicted binding free energy of in silico screening hits; Table S1: Ligand dataset used to generate three-dimensional pharmacophores. Physicochemical properties of the ligand dataset. MW: molecular weight; XLogP3-AA: octanol-water partition coefficient; HBD: hydrogen bond donor; HBA: hydrogen bond acceptor; RBC: rotatable bond count; TPSA: topological polar surface area; HAC: heavy atom count; IC50: half-maximal inhibitory concentration (nM); Table S2: In silico absorption, distribution, and toxicity prediction of natural compounds. HIA: human intestinal absorption; IVCCP: in vitro Caco-2 cell permeability; IVMCM: in vitro MDCK cell permeability; LogKp: in vitro skin permeability; IVPPB: in vitro plasma protein binding; BBP: in vivo blood–brain barrier penetration (C.brain/C.blood); Table S3: In silico absorption, distribution, and toxicity prediction of synthetic compounds. HIA: human intestinal absorption; IVCCP: in vitro Caco-2 cell permeability; IVMCM: in vitro MDCK cell permeability; LogKp: in vitro skin permeability; IVPPB: in vitro plasma protein binding; BBP: in vivo blood–brain barrier penetration (C.brain/C.blood); Video S1: Binding mode of virtually screened hit molecules.

## References

[1] Bogdanovic N., Hansson O., Zetterberg H., Basun H., Ingelsson M., Lannfelt L., Blennow K. (2020). Alzheimer’s disease—The most common cause of dementia. Lakartidningen.

[2] Marelli C., Hourregue C., Gutierrez L.A., Paquet C., de Menjot Champfleur N., De Verbizier D., Jacob M., Dubois J., Maleska A.M., Hirtz C. (2020). Cerebrospinal Fluid and Plasma Biomarkers do not Differ in the Presenile and Late-Onset Behavioral Variants of Frontotemporal Dementia. J. Alzheimers Dis..

[3] Wilson R.S., Segawa E., Boyle P.A., Anagnos S.E., Hizel L.P., Bennett D.A. (2012). The natural history of cognitive decline in Alzheimer’s disease. Psychol. Aging.

[4] Gaugler J., James B., Johnson T., Marin A., Weuve J., Assoc A.S. (2019). 2019 Alzheimer’s disease facts and figures. Alzheimers Dement..

[5] Gupta V., Gupta V.B., Chitranshi N., Gangoda S., Vander Wall R., Abbasi M., Golzan M., Dheer Y., Shah T., Avolio A. (2016). One protein, multiple pathologies: Multifaceted involvement of amyloid beta in neurodegenerative disorders of the brain and retina. Cell Mol. Life Sci..

[6] Gupta V.K., Chitranshi N., Gupta V.B., Golzan M., Dheer Y., Wall R.V., Georgevsky D., King A.E., Vickers J.C., Chung R. (2016). Amyloid beta accumulation and inner retinal degenerative changes in Alzheimer’s disease transgenic mouse. Neurosci. Lett..

[7] Zhang X., Fu Z., Meng L., He M., Zhang Z. (2018). The Early Events That Initiate beta-Amyloid Aggregation in Alzheimer’s Disease. Front. Aging Neurosci..

[8] Giorgetti S., Greco C., Tortora P., Aprile F.A. (2018). Targeting Amyloid Aggregation: An Overview of Strategies and Mechanisms. Int. J. Mol. Sci..

[9] Wang Q., Yu X., Li L., Zheng J. (2014). Inhibition of amyloid-beta aggregation in Alzheimer’s disease. Curr. Pharm. Des..

[10] Cavallo-Medved D., Moin K., Sloane B. (2011). Cathepsin B: Basis Sequence: Mouse. AFCS Nat. Mol. Pages.

[11] Linebaugh B.E., Sameni M., Day N.A., Sloane B.F., Keppler D. (1999). Exocytosis of active cathepsin B enzyme activity at pH 7.0, inhibition and molecular mass. Eur. J. Biochem..

[12] Liu C.L., Guo J., Zhang X., Sukhova G.K., Libby P., Shi G.P. (2018). Cysteine protease cathepsins in cardiovascular disease: From basic research to clinical trials. Nat. Rev. Cardiol..

[13] Gondi C.S., Rao J.S. (2013). Cathepsin B as a cancer target. Expert Opin. Ther. Targets.

[14] Sendler M., Maertin S., John D., Persike M., Weiss F.U., Kruger B., Wartmann T., Wagh P., Halangk W., Schaschke N. (2016). Cathepsin B Activity Initiates Apoptosis via Digestive Protease Activation in Pancreatic Acinar Cells and Experimental Pancreatitis. J. Biol. Chem..

[15] Bernstein H.G., Keilhoff G. (2018). Putative roles of cathepsin B in Alzheimer’s disease pathology: The good, the bad, and the ugly in one?. Neural. Regen. Res..

[16] Dewachter I., Van Leuven F. (2002). Secretases as targets for the treatment of Alzheimer’s disease: The prospects. Lancet Neurol..

[17] Yan R., Bienkowski M.J., Shuck M.E., Miao H., Tory M.C., Pauley A.M., Brashier J.R., Stratman N.C., Mathews W.R., Buhl A.E. (1999). Membrane-anchored aspartyl protease with Alzheimer’s disease beta-secretase activity. Nature.

[18] Lin X., Koelsch G., Wu S., Downs D., Dashti A., Tang J. (2000). Human aspartic protease memapsin 2 cleaves the beta-secretase site of beta-amyloid precursor protein. Proc. Natl. Acad. Sci. USA.

[19] Vassar R., Bennett B.D., Babu-Khan S., Kahn S., Mendiaz E.A., Denis P., Teplow D.B., Ross S., Amarante P., Loeloff R. (1999). Beta-secretase cleavage of Alzheimer’s amyloid precursor protein by the transmembrane aspartic protease BACE. Science.

[20] Mirzaei M., Pushpitha K., Deng L., Chitranshi N., Gupta V., Rajput R., Mangani A.B., Dheer Y., Godinez A., McKay M.J. (2019). Upregulation of Proteolytic Pathways and Altered Protein Biosynthesis Underlie Retinal Pathology in a Mouse Model of Alzheimer’s Disease. Mol. Neurobiol..

[21] Chitranshi N., Tiwari A.K., Somvanshi P., Tripathi P.K., Seth P.K. (2013). Investigating the function of single nucleotide polymorphisms in the CTSB gene: A computational approach. Futur. Neurol..

[22] Sosic I., Mitrovic A., Curic H., Knez D., Brodnik Zugelj H., Stefane B., Kos J., Gobec S. (2018). Cathepsin B inhibitors: Further exploration of the nitroxoline core. Bioorg. Med. Chem. Lett..

[23] Greenspan P.D., Clark K.L., Tommasi R.A., Cowen S.D., McQuire L.W., Farley D.L., van Duzer J.H., Goldberg R.L., Zhou H., Du Z. (2001). Identification of dipeptidyl nitriles as potent and selective inhibitors of cathepsin B through structure-based drug design. J. Med. Chem..

[24] Rasnick D. (1985). Synthesis of peptide fluoromethyl ketones and the inhibition of human cathepsin B. Anal. Biochem..

[25] Jilkova A., Rezacova P., Lepsik M., Horn M., Vachova J., Fanfrlik J., Brynda J., McKerrow J.H., Caffrey C.R., Mares M. (2011). Structural basis for inhibition of cathepsin B drug target from the human blood fluke, Schistosoma mansoni. J. Biol. Chem..

[26] Zhou Z., Wang Y., Bryant S.H. (2009). Computational analysis of the cathepsin B inhibitors activities through LR-MMPBSA binding affinity calculation based on docked complex. J. Comput. Chem..

[27] Perlman N., Hazan M., Shokhen M., Albeck A. (2008). Peptidyl epoxides extended in the P’ direction as cysteine protease inhibitors: Effect on affinity and mechanism of inhibition. Bioorg. Med. Chem..

[28] Siklos M., BenAissa M., Thatcher G.R. (2015). Cysteine proteases as therapeutic targets: Does selectivity matter? A systematic review of calpain and cathepsin inhibitors. Acta Pharm. Sin. B.

[29] Walker B., McCarthy N., Healy A., Ye T., McKervey M.A. (1993). Peptide glyoxals: A novel class of inhibitor for serine and cysteine proteinases. Biochem. J..

[30] Schaschke N., Deluca D., Assfalg-Machleidt I., Hohneke C., Sommerhoff C.P., Machleidt W. (2002). Epoxysuccinyl peptide-derived cathepsin B inhibitors: Modulating membrane permeability by conjugation with the C-terminal heptapeptide segment of penetratin. Biol. Chem..

[31] Schmitz J., Li T., Bartz U., Gutschow M. (2016). Cathepsin B Inhibitors: Combining Dipeptide Nitriles with an Occluding Loop Recognition Element by Click Chemistry. ACS Med. Chem. Lett..

[32] Pan X., Tan N., Zeng G., Zhang Y., Jia R. (2005). Amentoflavone and its derivatives as novel natural inhibitors of human Cathepsin B. Bioorg. Med. Chem..

[33] Turk D., Podobnik M., Popovic T., Katunuma N., Bode W., Huber R., Turk V. (1995). Crystal structure of cathepsin B inhibited with CA030 at 2.0-A resolution: A basis for the design of specific epoxysuccinyl inhibitors. Biochemistry.

[34] Berman H.M., Westbrook J., Feng Z., Gilliland G., Bhat T.N., Weissig H., Shindyalov I.N., Bourne P.E. (2000). The Protein Data Bank. Nucleic. Acids. Res..

[35] Chitranshi N., Gupta V.K., Rajput R., Godinez A., Pushpitha K., Shen T., Mirzaei M., You Y., Basavarajappa D., Gupta V. (2020). Evolving geographic diversity in SARS-CoV2 and in silico analysis of replicating enzyme 3CL(pro) targeting repurposed drug candidates. J. Transl. Med..

[36] Pettersen E.F., Goddard T.D., Huang C.C., Couch G.S., Greenblatt D.M., Meng E.C., Ferrin T.E. (2004). UCSF Chimera--a visualization system for exploratory research and analysis. J. Comput. Chem..

[37] Kim S., Chen J., Cheng T., Gindulyte A., He J., He S., Li Q., Shoemaker B.A., Thiessen P.A., Yu B. (2019). PubChem 2019 update: Improved access to chemical data. Nucleic. Acids. Res..

[38] Zeng G.Z., Pan X.L., Tan N.H., Xiong J., Zhang Y.M. (2006). Natural biflavones as novel inhibitors of cathepsin B and K. Eur. J. Med. Chem..

[39] Powers J.C., Asgian J.L., Ekici O.D., James K.E. (2002). Irreversible inhibitors of serine, cysteine, and threonine proteases. Chem. Rev..

[40] Sarabia F., Sanchez-Ruiz A., Chammaa S. (2005). Stereoselective synthesis of E-64 and related cysteine proteases inhibitors from 2,3-epoxyamides. Bioorg. Med. Chem..

[41] Morris G.M., Goodsell D.S., Halliday R.S., Huey R., Hart W.E., Belew R.K., Olson A.J. (1998). Automated docking using a Lamarckian genetic algorithm and an empirical binding free energy function. J. Comput. Chem..

[42] Chitranshi N., Gupta S., Tripathi P.K., Seth P.K. (2013). New molecular scaffolds for the design of Alzheimer’s acetylcholinesterase inhibitors identified using ligand- and receptor-based virtual screening. Med. Chem. Res..

[43] Chitranshi N., Gupta V., Dheer Y., Gupta V., Vander Wall R., Graham S. (2016). Molecular determinants and interaction data of cyclic peptide inhibitor with the extracellular domain of TrkB receptor. Data Brief..

[44] Xiao J., Zhang S., Luo M., Zou Y., Zhang Y., Lai Y. (2015). Effective virtual screening strategy focusing on the identification of novel Bruton’s tyrosine kinase inhibitors. J. Mol. Graph. Model..

[45] Wolber G., Langer T. (2005). LigandScout: 3-D pharmacophores derived from protein-bound ligands and their use as virtual screening filters. J. Chem. Inf. Model..

[46] Trott O., Olson A.J. (2010). AutoDock Vina: Improving the speed and accuracy of docking with a new scoring function, efficient optimization, and multithreading. J. Comput. Chem..

[47] Truchon J.F., Bayly C.I. (2007). Evaluating virtual screening methods: Good and bad metrics for the “early recognition” problem. J. Chem. Inf. Model..

[48] Benet L.Z., Hosey C.M., Ursu O., Oprea T.I. (2016). BDDCS, the Rule of 5 and drugability. Adv. Drug Deliv. Rev..

[49] Gupta S., Misra G., Chandra Pant M., Kishore Seth P. (2012). Targeting the epidermal growth factor receptor: Exploring the potential of novel inhibitor N-(3-ethynylphenyl)-6, 7-bis (2-methoxyethoxy) quinolin- 4-amine using docking and molecular dynamics simulation. Protein Pept. Lett..

[50] Kumar A., Siddiqi M.I. (2008). Virtual screening against Mycobacterium tuberculosis dihydrofolate reductase: Suggested workflow for compound prioritization using structure interaction fingerprints. J. Mol. Graph. Model..

[51] Velazquez-Libera J.L., Murillo-Lopez J.A., de la Torre A.F., Caballero J. (2019). Structural Requirements of N-alpha-Mercaptoacetyl Dipeptide (NAMdP) Inhibitors of Pseudomonas Aeruginosa Virulence Factor LasB: 3D-QSAR, Molecular Docking, and Interaction Fingerprint Studies. Int. J. Mol. Sci..

[52] Racz A., Bajusz D., Heberger K. (2018). Life beyond the Tanimoto coefficient: Similarity measures for interaction fingerprints. J. Cheminform..

[53] Bocker A., Derksen S., Schmidt E., Teckentrup A., Schneider G. (2005). A hierarchical clustering approach for large compound libraries. J. Chem. Inf. Model..

[54] Ji B., Liu S., He X., Man V.H., Xie X.Q., Wang J. (2020). Prediction of the Binding Affinities and Selectivity for CB1 and CB2 Ligands Using Homology Modeling, Molecular Docking, Molecular Dynamics Simulations, and MM-PBSA Binding Free Energy Calculations. ACS Chem. Neurosci..

[55] Genheden S., Ryde U. (2015). The MM/PBSA and MM/GBSA methods to estimate ligand-binding affinities. Expert Opin. Drug. Discov..

[56] Zhang X., Perez-Sanchez H., Lightstone F.C. (2017). A Comprehensive Docking and MM/GBSA Rescoring Study of Ligand Recognition upon Binding Antithrombin. Curr. Top. Med. Chem..

[57] Yokoyama M., Fujisaki S., Shirakura M., Watanabe S., Odagiri T., Ito K., Sato H. (2017). Molecular Dynamics Simulation of the Influenza A(H3N2) Hemagglutinin Trimer Reveals the Structural Basis for Adaptive Evolution of the Recent Epidemic Clade 3C.2a. Front. Microbiol..

[58] Chitranshi N., Dheer Y., Kumar S., Graham S.L., Gupta V. (2019). Molecular docking, dynamics, and pharmacology studies on bexarotene as an agonist of ligand-activated transcription factors, retinoid X receptors. J. Cell Biochem..

[59] Boateng H.A. (2020). Periodic Coulomb Tree Method: An Alternative to Parallel Particle Mesh Ewald. J. Chem. Theory Comput..

[60] Elber R., Ruymgaart A.P., Hess B. (2011). SHAKE parallelization. Eur. Phys. J. Spec. Top..

[61] Case D.A., Cheatham T.E., Darden T., Gohlke H., Luo R., Merz K.M., Onufriev A., Simmerling C., Wang B., Woods R.J. (2005). The Amber biomolecular simulation programs. J. Comput. Chem..

[62] Onufriev A.V., Case D.A. (2019). Generalized Born Implicit Solvent Models for Biomolecules. Annu. Rev. Biophys..

[63] Huang H., Simmerling C. (2018). Fast Pairwise Approximation of Solvent Accessible Surface Area for Implicit Solvent Simulations of Proteins on CPUs and GPUs. J. Chem. Theory Comput..

[64] Chitranshi N., Gupta V., Kumar S., Graham S.L. (2015). Exploring the Molecular Interactions of 7,8-Dihydroxyflavone and Its Derivatives with TrkB and VEGFR2 Proteins. Int. J. Mol. Sci..

[65] Lipinski C.A., Lombardo F., Dominy B.W., Feeney P.J. (2001). Experimental and computational approaches to estimate solubility and permeability in drug discovery and development settings. Adv. Drug Deliv. Rev..

[66] Chetter B.A., Kyriakis E., Barr D., Karra A.G., Katsidou E., Koulas S.M., Skamnaki V.T., Snape T.J., Psarra A.G., Leonidas D.D. (2020). Synthetic flavonoid derivatives targeting the glycogen phosphorylase inhibitor site: QM/MM-PBSA motivated synthesis of substituted 5,7-dihydroxyflavones, crystallography, in vitro kinetics and ex-vivo cellular experiments reveal novel potent inhibitors. Bioorg. Chem..

[67] Ghosh R., Chakraborty A., Biswas A., Chowdhuri S. (2020). Identification of polyphenols from Broussonetia papyrifera as SARS CoV-2 main protease inhibitors using in silico docking and molecular dynamics simulation approaches. J. Biomol. Struct. Dyn..

[68] El Khoury L., Santos-Martins D., Sasmal S., Eberhardt J., Bianco G., Ambrosio F.A., Solis-Vasquez L., Koch A., Forli S., Mobley D.L. (2019). Comparison of affinity ranking using AutoDock-GPU and MM-GBSA scores for BACE-1 inhibitors in the D3R Grand Challenge 4. J. Comput. Aided. Mol. Des..

[69] Wang E., Weng G., Sun H., Du H., Zhu F., Chen F., Wang Z., Hou T. (2019). Assessing the performance of the MM/PBSA and MM/GBSA methods. 10. Impacts of enhanced sampling and variable dielectric model on protein-protein Interactions. Phys. Chem. Chem. Phys..

[70] Joseph C., Mangani A.S., Gupta V., Chitranshi N., Shen T., Dheer Y., Kb D., Mirzaei M., You Y., Graham S.L. (2020). Cell Cycle Deficits in Neurodegenerative Disorders: Uncovering Molecular Mechanisms to Drive Innovative Therapeutic development. Aging Dis..

[71] Gupta V.B., Chitranshi N., Haan J.D., Mirzaei M., You Y., Lim J.K., Basavarajappa D., Godinez A., Di Angelantonio S., Sachdev P. (2020). Retinal changes in Alzheimer’s disease- integrated prospects of imaging, functional and molecular advances. Prog. Retin. Eye. Res..

[72] Morris G.P., Clark I.A., Vissel B. (2014). Inconsistencies and controversies surrounding the amyloid hypothesis of Alzheimer’s disease. Acta Neuropathol. Commun..

[73] Simic G., Babic Leko M., Wray S., Harrington C., Delalle I., Jovanov-Milosevic N., Bazadona D., Buee L., de Silva R., Di Giovanni G. (2016). Tau Protein Hyperphosphorylation and Aggregation in Alzheimer’s Disease and Other Tauopathies, and Possible Neuroprotective Strategies. Biomolecules.

[74] Hook G., Hook V., Kindy M. (2011). The cysteine protease inhibitor, E64d, reduces brain amyloid-beta and improves memory deficits in Alzheimer’s disease animal models by inhibiting cathepsin B, but not BACE1, beta-secretase activity. J. Alzheimers Dis..

[75] Sakr M.F., Hassanein T.I., Zetti G.M., Van Thiel D.H. (1990). FK 506 ameliorates the hepatic injury associated with ischemia. Life Sci..

[76] Wang C., Sun B., Zhou Y., Grubb A., Gan L. (2012). Cathepsin B degrades amyloid-beta in mice expressing wild-type human amyloid precursor protein. J. Biol. Chem..

[77] Embury C.M., Dyavarshetty B., Lu Y., Wiederin J.L., Ciborowski P., Gendelman H.E., Kiyota T. (2017). Cathepsin B Improves ss-Amyloidosis and Learning and Memory in Models of Alzheimer’s Disease. J Neuroimmune Pharm..

[78] Smith A.J., Zhang X., Leach A.G., Houk K.N. (2009). Beyond picomolar affinities: Quantitative aspects of noncovalent and covalent binding of drugs to proteins. J. Med. Chem..

[79] Katritch V., Byrd C.M., Tseitin V., Dai D., Raush E., Totrov M., Abagyan R., Jordan R., Hruby D.E. (2007). Discovery of small molecule inhibitors of ubiquitin-like poxvirus proteinase I7L using homology modeling and covalent docking approaches. J. Comput. Aided. Mol. Des..

[80] Khan M.S., Mehmood B., Yousafi Q., Bibi S., Fazal S., Saleem S., Sajid M.W., Ihsan A., Azhar M., Kamal M.A. (2021). Molecular Docking studies reveals Rhein from rhubarb (Rheum rhabarbarum) as a putative inhibitor of ATP-binding Cassette Super Family G member 2. Med. Chem..

[81] Dhanjal J.K., Sharma S., Grover A., Das A. (2015). Use of ligand-based pharmacophore modeling and docking approach to find novel acetylcholinesterase inhibitors for treating Alzheimer’s. Biomed. Pharm..

[82] Gimeno A., Ojeda-Montes M.J., Tomas-Hernandez S., Cereto-Massague A., Beltran-Debon R., Mulero M., Pujadas G., Garcia-Vallve S. (2019). The Light and Dark Sides of Virtual Screening: What is There to Know?. Int. J. Mol. Sci..

[83] Kutlushina A., Khakimova A., Madzhidov T., Polishchuk P. (2018). Ligand-Based Pharmacophore Modeling Using Novel 3D Pharmacophore Signatures. Molecules.

[84] Chandra N., Bhagavat R., Sharma E., Sreekanthreddy P., Somasundaram K. (2011). Virtual screening, identification and experimental testing of novel inhibitors of PBEF1/Visfatin/NMPRTase for glioma therapy. J. Clin. Bioinform..

[85] Fu Y., Zhao J., Chen Z. (2018). Insights into the Molecular Mechanisms of Protein-Ligand Interactions by Molecular Docking and Molecular Dynamics Simulation: A Case of Oligopeptide Binding Protein. Comput. Math. Methods Med..

[86] Liu H., Wang L., Lv M., Pei R., Li P., Pei Z., Wang Y., Su W., Xie X.Q. (2014). AlzPlatform: An Alzheimer’s disease domain-specific chemogenomics knowledgebase for polypharmacology and target identification research. J. Chem. Inf. Model..

[87] Nogara P.A., Saraiva Rde A., Caeran Bueno D., Lissner L.J., Lenz Dalla Corte C., Braga M.M., Rosemberg D.B., Rocha J.B. (2015). Virtual screening of acetylcholinesterase inhibitors using the Lipinski’s rule of five and ZINC databank. Biomed. Res. Int..

[88] Lionta E., Spyrou G., Vassilatis D.K., Cournia Z. (2014). Structure-based virtual screening for drug discovery: Principles, applications and recent advances. Curr. Top. Med. Chem..

[89] Fuhrmann J., Rurainski A., Lenhof H.P., Neumann D. (2010). A new Lamarckian genetic algorithm for flexible ligand-receptor docking. J. Comput. Chem..

[90] Ritschel T., Schirris T.J., Russel F.G. (2014). KRIPO—A structure-based pharmacophores approach explains polypharmacological effects. J. Cheminform..

[91] Loser R., Pietzsch J. (2015). Cysteine cathepsins: Their role in tumor progression and recent trends in the development of imaging probes. Front. Chem..

[92] Hospital A., Goni J.R., Orozco M., Gelpi J.L. (2015). Molecular dynamics simulations: Advances and applications. Adv. Appl. Bioinform. Chem..

[93] Durrant J.D., McCammon J.A. (2011). Molecular dynamics simulations and drug discovery. BMC Biol..

[94] Gohlke H., Klebe G. (2002). Approaches to the description and prediction of the binding affinity of small-molecule ligands to macromolecular receptors. Angew. Chem. Int. Ed. Engl..

[95] Wang Y.Q., Lin W.W., Wu N., Wang S.Y., Chen M.Z., Lin Z.H., Xie X.Q., Feng Z.W. (2019). Structural insight into the serotonin (5-HT) receptor family by molecular docking, molecular dynamics simulation and systems pharmacology analysis. Acta Pharmacol. Sin..

[96] Mazumder M., Ponnan P., Das U., Gourinath S., Khan H.A., Yang J., Sakharkar M.K. (2017). Investigations on Binding Pattern of Kinase Inhibitors with PPARgamma: Molecular Docking, Molecular Dynamic Simulations, and Free Energy Calculation Studies. PPAR Res..

[97] Taddei M., Ferrini S., Giannotti L., Corsi M., Manetti F., Giannini G., Vesci L., Milazzo F.M., Alloatti D., Guglielmi M.B. (2014). Synthesis and evaluation of new Hsp90 inhibitors based on a 1,4,5-trisubstituted 1,2,3-triazole scaffold. J. Med. Chem..

[98] Roca C., Martinez-Gonzalez L., Daniel-Mozo M., Sastre J., Infantes L., Mansilla A., Chaves-Sanjuan A., Gonzalez-Rubio J.M., Gil C., Canada F.J. (2018). Deciphering the Inhibition of the Neuronal Calcium Sensor 1 and the Guanine Exchange Factor Ric8a with a Small Phenothiazine Molecule for the Rational Generation of Therapeutic Synapse Function Regulators. J. Med. Chem..

[99] Koukoulitsa C., Villalonga-Barber C., Csonka R., Alexi X., Leonis G., Dellis D., Hamelink E., Belda O., Steele B.R., Micha-Screttas M. (2016). Biological and computational evaluation of resveratrol inhibitors against Alzheimer’s disease. J. Enzyme. Inhib. Med. Chem..

[100] Sharma T., Harioudh M.K., Kuldeep J., Kumar S., Banerjee D., Ghosh J.K., Siddiqi M.I. (2020). Identification of Potential Inhibitors of Cathepsin-B using Shape & Pharmacophore-based Virtual Screening, Molecular Docking and Explicit Water Thermodynamics. Mol. Inform..

