# Supplementary material for: Identification of Novel Cathepsin B Inhibitors with Implications in Alzheimer’s Disease: Computational Refining and Biochemical Evaluation
Source: Cells. 2021 Jul 31;10(8):1946. doi: 10.3390/cells10081946 (PMC8391575; doi:10.3390/cells10081946)
Supplement: Supplementary file 1 [file cells-10-01946-s001.zip › cells-1295006 supplementary proofreading back/Supplementary/Supplementary Files.pdf]

Identification of novel cathepsin B inhibitors with implications in Alzheimer's disease: Computational refining and biochemical evaluation

Nitin Chitranshi [1,*], Ashutosh Kumar [2], Samran Sheriff [1], Veer Gupta [3], Angela Godinez [1], Danit Saks [1], Soumalya Sarkar [1], Ting Shen [1], Mehdi Mirzaei [1], Devaraj Basavarajappa [1], Morteza Abyadeh [4], Sachin K Singh [5], Kamal Dua [6,7], Kam Y. J. Zhang [2], Stuart L. Graham [1] and Vivek Gupta [1,*]

[1] Faculty of Medicine, Health and Human Sciences, Macquarie University, F10A, 2 Technology Place, North Ryde, NSW 2109, Australia; samran.sheriff@hdr.mq.edu.au (S.S.); angela.godinez@hdr.mq.edu.au (A.G.); danit.saks@hdr.mq.edu.au (D.S.); soumalya.sarkar@hdr.mq.edu.au (S.S.); ting.shen@mq.edu.au (T.S.); mehdi.mirzaei@mq.edu.au (M.M.); devaraj.basavarajappa@mq.edu.au (D.B.); stuart.graham@mq.edu.au (S.L.G.)

[2] Laboratory for Structural Bioinformatics, Center for Biosystems Dynamics Research, RIKEN, 1-7-22 Suehiro, Tsurumi, Yokohama 230-0045, Kanagawa, Japan; akumar@riken.jp (A.K.); kamzhang@riken.jp (K.Y.J.Z.)

[3] School of Medicine, Faculty of Health, Deakin University, Geelong, Victoria, 3220, Australia; veer.gupta@deakin.edu.au

[4] Cell Science Research Center, Department of Molecular Systems Biology, Royan Institute for Stem Cell Biology and Technology, ACECR, Tehran 1665659911, Iran; mabyadeh@yahoo.com

[5] School of Pharmaceutical Sciences, Lovely Professional University, Phagwara, Punjab 144411, India; sachin.16030@lpu.co.in

[6] Discipline of Pharmacy, Graduate School of Health, University of Technology Sydney, Ultimo NSW 2007, Australia; kamal.dua@uts.edu.au

[7] Faculty of Health, Australian Research Centre in Complementary and Integrative Medicine, University of Technology Sydney, Ultimo NSW 2007, Australia

* Correspondence: nitin.chitranshi@mq.edu.au (N.C.); vivek.gupta@mq.edu.au (V.G.); Tel.: +61 (02) 9850 2804

Table S1. : Ligand Dataset used to generate three dimensional pharmacophore. Physico-chemical properties of ligand dataset. MW, molecular weight; XLogP3-AA, octanol-water partition coefficient; HBD, hydrogen bond donor; HBA, hydrogen bond acceptor; RBC, rotatable bond count; TPSA: topological polar surface area; HAC, heavy atom count; IC_{50}: half maximal inhibitory concentration (µM).

NATURAL COMPOUNDS

S. No.	Name	IUPAC Name	M/W	XLogP3-AA	HBD	HBA	RBC	TPSA	HAC	IC50	Structure
N1*	AM 4299 A	3-[[1-(5-hydroxypentylamino)-4-methyl-1-oxopentan-2-yl]carbamoyl]oxirane-2-carboxylic acid	330.3767	0.3	4	6	11	128	23	0.073	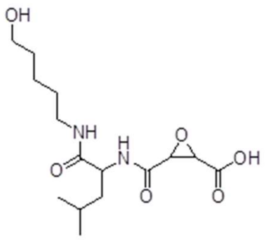
N2*	AM 4299 B	3-[[(2S)-1-[[(5S)-5-amino-5-carboxypentyl]amino]-4-methyl-1-oxopentan-2-yl]carbamoyl]oxirane-2-carboxylic acid	373.4014	-2.6	5	8	12	171	26	0.13	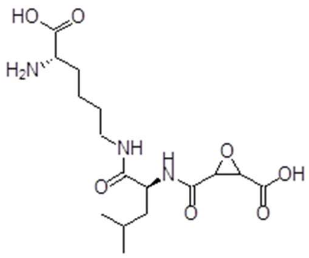
N3	Amentoflavone (AMF 1)	8-[5-(5,7-dihydroxy-4-oxochromen-2-yl)-2-hydroxyphenyl]-5,7-dihydroxy-2-(4-hydroxyphenyl)chromen-4-one	538.4579	5	6	10	3	174	40	1.75	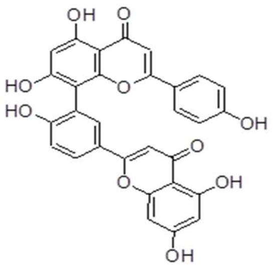
	AMF 4/										

N4*	Amentoflavone-4',4'',7-trimethyl Ether/ sciadopitysin	5,7-dihydroxy-8-[5-(5-hydroxy-7-methoxy-4-oxochromen-2-yl)-2-methoxyphenyl]-2-(4-methoxyphenyl)chromen-4-one	580.5376	6	3	10	6	141	43	0.62	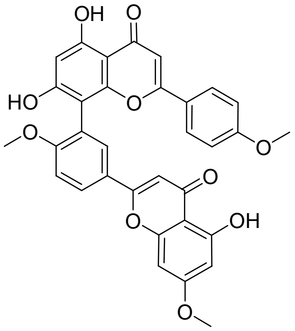
N5	Antipain Dihydrochloride	2-[[5-(diaminomethylideneamino)-1-[[1-[[5-(diaminomethylideneamino)-1-oxopentan-2-yl]amino]-3-methyl-1-oxobutan-2-yl]amino]-1-oxopentan-2-yl] carbamoylamino]-3-phenylpropanoic acid dihydrochloride	677.6235	-3.009	11	8	19	283	45	0.59	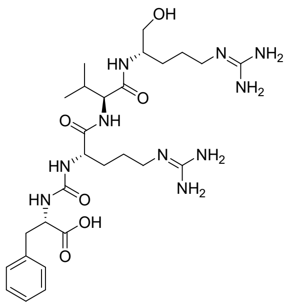

N6 *	Cath es- tatin A	(2S,3S)-3-({(2S)-1-[(4-Aminobu- tyl)amino]-1- oxo-3-phenyl- 2-propa- nyl}car- bamoyl)-2- oxiranecar- boxylic acid	34 9.1 63 7	-1.3	5	8	11	134	25	0.26	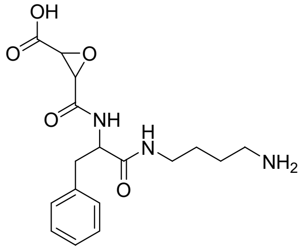
N7 *	Cath es- tatin B	(2S,3S)-3- {[(2S)-1-[(4- Aminobu- tyl)amino]-3- (4-hydroxy- phenyl)-1- oxo-2-propa- nyl]car- bamoyl}-2- oxiranecar- boxylic acid	36 5.1 58 6	- 0.24 3	6	9	12	154	26	0.28	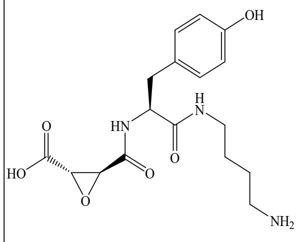
N8	Cela strol	(2R,4aS,6aR,6a S,14aS,14bR)- 10-hydroxy- 2,4a,6a,6a,9,14 a-hexamethyl- 11-oxo 1,3,4,5,6,13,14, 14b-octahy- dropicene-2- carboxylic acid	45 0.6 09 6	5.9	2	4	1	74. 6	33	1.25	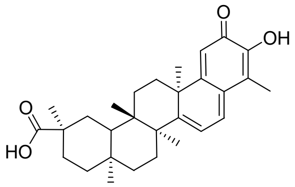
N9		(2S)-2-[[(1S)-1- (2-amino- 1,4,5,6-tetra- hydropyrim- idin-6-yl)-2- [[(2S)-4-		1.8	7	7	15	204	44	2.6	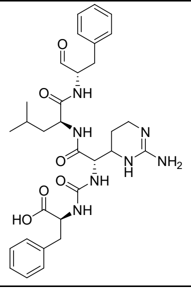

	Chymostatin	methyl-1-oxo-1-[[(2S)-1-oxo-3-phenylpropan-2-yl]amino]pentan-2-yl]amino]-2-oxoethyl]carbamoylamino]-3-phenylpropanoic acid	607.7005								
N10*	CP-1 (Imidacloprid)	N-[1-[(6-chloropyridin-3-yl)methyl]-4,5-dihydroimidazol-2-yl]nitramide	255.661	0.8	1	4	3	86.3	17	0.0094	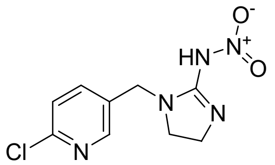
N11	CP-3	5,10-bis(1-methylpyridin-1-ium-3-yl)-15,20-diphenyl-21,22-dihydroporphyrin	646.7809	8.5	2	2	4	65.1	50	0.0008	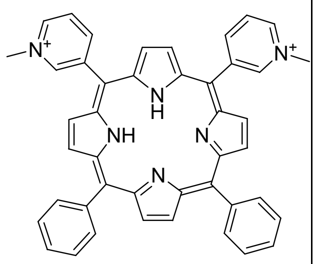
N12	CP-5 (Ellipticine)	5,11-dimethyl-6H-pyrido[4,3-b]carbazole	246.3064	4.8	1	1	0	28.7	19	0.0103	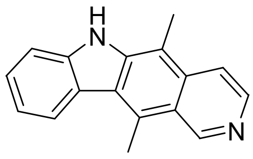
	E-64	(2S,3S)-3-[[(2S)-1-[4-	357.4	-0.9	5	6	11	172	25		

N13*		(diaminomethylideneamino)butylamino]-4-methyl-1-oxopentan-2-yl]carbamoyl]oxirane-2-carboxylic acid	053							0.0052	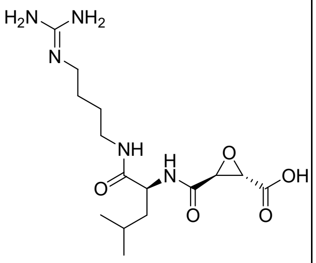
N14*	E-64c	(2S,3S)-3-[[(2S)-4-methyl-1-(3-methylbutylamino)-1-oxopentan-2-yl]carbamoyl]oxirane-2-carboxylic acid	314.3773	1.6	3	5	9	108	22	0.0034	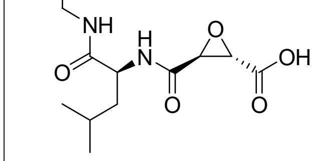
N15*	Estatin A	3-[[1-[4-(diaminomethylideneamino)butylamino]-1-oxo-3-phenylpropan-2-yl]carbamoyl]oxirane-2-carboxylic acid	391.4216	-0.6	5	6	11	172	28	0.27	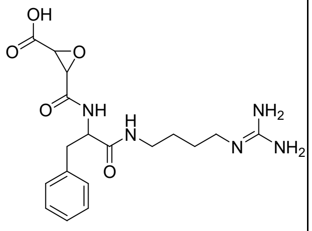
N16	Estatin B	3-[[1-[4-(diaminomethylideneamino)butylamino]-3-(4-hydroxyphenyl)-	407.421	-1	6	7	11	193	29	0.32	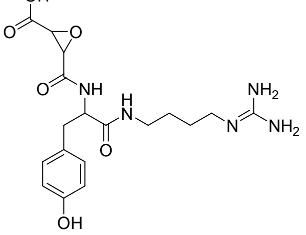

		1-oxopropan-2-yl]carbamoyl]oxirane-2-carboxylic acid									
N17*	Ginkgetin / AMF 3	5,7-dihydroxy-8-[5-(5-hydroxy-7-methoxy-4-oxochromen-2-yl)-2-methoxyphenyl]-2-(4-hydroxyphenyl)chromen-4-one	566.5110	5.7	4	10	5	152	42	0.55	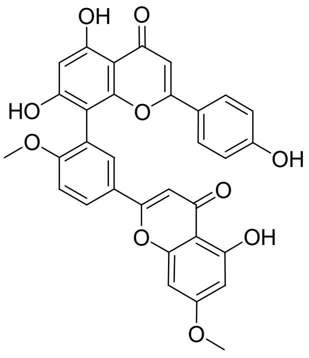
N18	HIF-1alpha (hypoxia-inducible factor 1, alpha)	1-[2-amino-6-(3,5-dimethoxyphenyl)pyrido[2,3-d]pyrimidin-7-yl]-3-tert-butylurea	396.4429	2.3	3	3	5	124	29	0.58	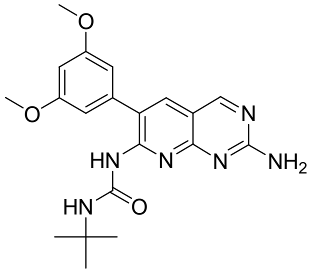

N19	Leupeptin trifluoroacetate	2-acetamido-N-[1-[[5-(diaminomethylideneamino)-1-oxopentan-2-yl]amino]-4-methyl-1-oxopentan-2-yl]-4-methylpentanamide; 2,2,2-trifluoroacetic acid	540.5768	0.032	6	10	14	206	37	0.44	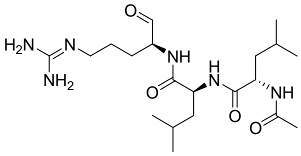
N20*	Loxistatin (E-64d)	ethyl (2S,3S)-3-[[(2S)-4-methyl-1-(3-methylbutylamino)-1-oxopentan-2-yl]carbamoyl]oxirane-2-carboxylate	342.4305	2.3	2	5	11	97	24	0.0034	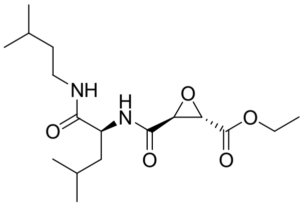
N21	Sequoiaflavone (AMF 2)	5,7-dihydroxy-8-[2-hydroxy-5-(5-hydroxy-7-methoxy-4-oxochromen-2-yl)phenyl]-2-(4-hydroxyphenyl)chromen-4-one	552.4845	5.4	5	10	4	163	41	1.68	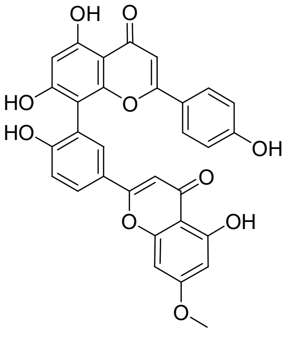
N22		1,6-dimethylpyrimido[5,4-	193.1	-0.7	0	3	0	77.7	14	0.046	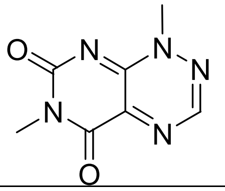

	Tox ofla-vin	e][1,2,4]tria-zine-5,7-dione	62 7								

SYNTHETIC COMPOUNDS

S1*	CA-074	(2S)-1-[(2S,3S)-3-methyl-2-[[(3S)-3-(propylcar-bamoyl)oxirane-2-car-bonyl]amino]penta-noyl]pyr-rolidine-2-carboxylic acid	38 3.4 39 3	0.9	3	6	9	12 8	27	0.00 2	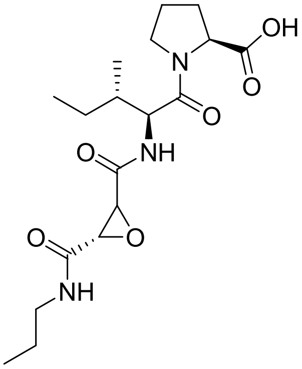
		(2S)-1-[(2S,3S)-2-[[(2S,3S)-3-									

S2*	CA-030	ethoxycarbonyloxirane-2-carbonyl]amino]-3-methylpentanoyl] pyrrolidine-2-carboxylic acid	370.3975	0.9	2	7	9	126	26	0.0028	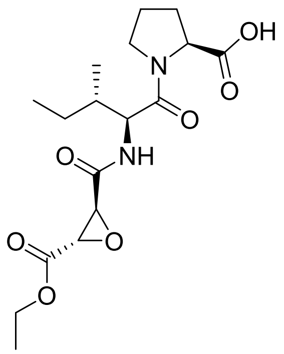
S3*	Cathepsin B Inhibitor II (acetyl-L-leucyl-L-valyl-L-lysinal)	2-acetamido-N-[1-[(6-amino-1-oxohexan-2-yl)amino]-3-methyl-1-oxobutan-2-yl]-4-methylpentanamide	384.5135	0.4	4	5	13	130	27	0.004	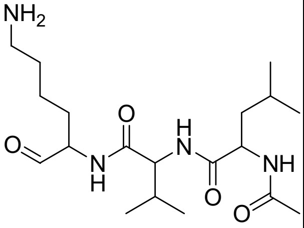
S4*	Z-Phe-Ala-diazomethylketone	1-diazonio-3-[[3-phenyl-2-(phenylmethoxycarbonylamino)propanoyl]amino]but-1-en-2-olate	394.4237	4.2	2	5	9	119	29	0.0094	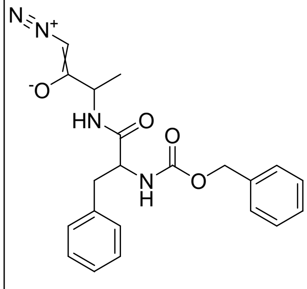
S5	4-[1-(4-Fluorobenzyl)-		309.2	2.3	1	4	3		23		

	1H-benzoimidazol-2-yl]-furazan-3-ylamine	4-[1-[(4-fluorophenyl)methyl]benzimidazol-2-yl]-1,2,5-oxadiazol-3-amine	977					82.8		33.85	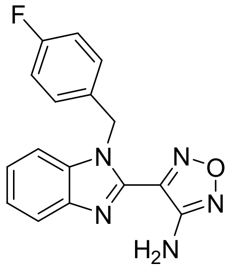
S6	Furazan, di-p-anisoyl-, 2-oxide	[4-(4-methoxybenzoyl)-5-oxido-1,2,5-oxadiazol-5-ium-3-yl]-(4-methoxyphenyl)methanone	354.3135	3.3	0	7	6	104	26	11.45	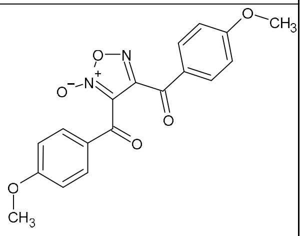
S7	ZINC00812573	[5-amino-1-(4-methylphenyl)sulfonylpyrazol-3-yl] thiophene-2-carboxylate	363.4114	3.8	1	6	5	141	24	1.99	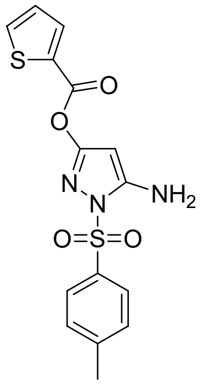
S8	T6703041	5-amino-1-(4-	387.4	3.7	1	6	6	122	27	12.26	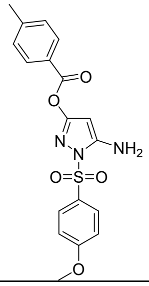

		methoxy-phenyl)sul-fonylpyra-zol-3-yl] 4-methylben-zoate	096								
S9*	MLS000027832	1-ethyl-6-methyl-3-phenylpy-rimido[5,4-e][1,2,4]tri-azine-5,7-dione	283.2853	1.4	0	3	2	77.7	21	0.0071	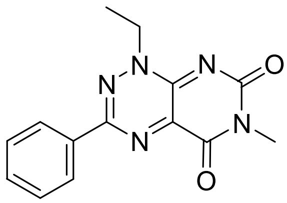
S10	SMR000014918	[5-amino-1-(4-fluoro-phenyl)sul-fonylpyra-zol-3-yl] furan-2-carboxylate	351.3097	2.9	1	7	5	126	24	1.261	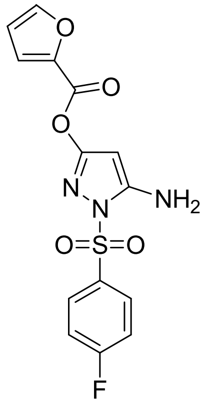
S11		[2-oxo-1-pyridin-2-yl-2-									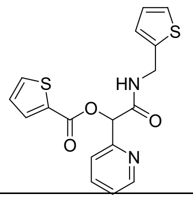

	SMR00000803 6	(thiophen-2-ylmethyl-a-mino)ethyl]thiophene-2-carbox-ylate	358.4346	3	1	6	7	125	24	6.356	
S12	Furan-2-car-boxylic acid 5-amino-1-(tolu-ene-4-sul-fonyl)-1H-py-razol-3-yl ester	[5-amino-1-(4-methylphe nyl)sul-fonylpyra-zol-3-yl] furan-2-carboxylate	347.3458	3.2	1	6	5	126	24	1.75	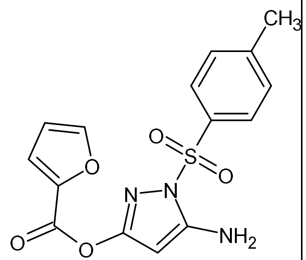
S13 *	1-Ethyl-6-me-thyl-3-thio-phen-2-yl-1H-pyrim-ido[5,4-e][1,2,4]tria-zine-5,7-di-one	1-Ethyl-6-methyl-3-thiophen-2-yl-1H-py-rimido[5,4-e][1,2,4]tri-azine-5,7-dione	289.3130	1.4	0	4	2	106	20	0.072	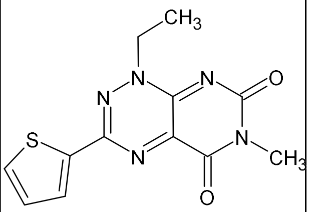
S14	2-[2-(4-Amino-fura-zan-3-yl)-ben-zoimid-azol-1-	2-[2-(4-Amino-furazan-3-yl)-benzo-imidazol-1-	326.3531	1.1	1	4	3	103	24	44.57	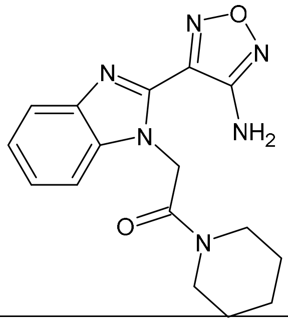

	yl]-1-piperidin-1-yl-ethanone	yl]-1-piperidin-1-yl-ethanone									
S15*	SMR000014916	5-amino-1-(4-methoxyphenyl)sulfonylpyrazol-3-yl] furan-2-carboxylate	363.3452	2.8	1	7	6	135	25	0.924	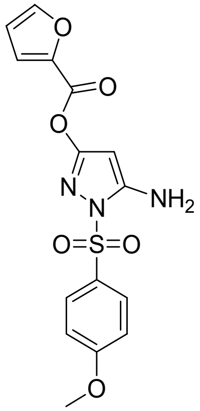
S16	1-Acetyl-3-(3,4-dichloro-5-oxo-2,5-dihydrofuran-2-yl)-urea	N-[(3,4-dichloro-5-oxo-2H-furan-2-yl)carbamoyl]acetamide	253.0395	1	2	4	1	84.5	15	2.12	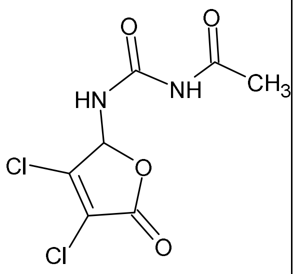
S17	T6771748	[5-amino-1-(4-methoxyphenyl)sulfonylpyrazol-3-yl] benzoate	373.3831	3.4	1	6	6	122	26	9.563	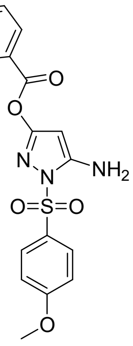
		methyl(5-cyano-3,3-									

S18	SMR00003902 2	dimethyl-8-morpholin-4-yl-1,4-di-hydropy-rano[3,4-c]pyridin-6-yl)sul-fanylfor-mate	363.4313	2	0	7	4	110	25	19.986	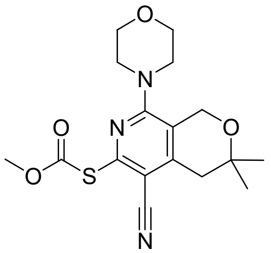
S19	SMR00003905 3	diethyl 2-[cyano-[4-(dime-thyla-mino)-6-methyl-sulfanyl-1,3, 5-triazin-2-yl]amino]p ropanedi-oate	368.4114	2.6	0	6	10	147	25	6.715	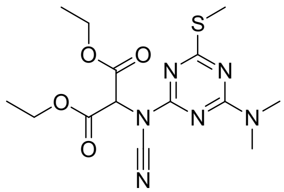
S20	SMR00003973 4	[4-[(2-methoxy-phenyl)imi nomethyl]-2-phenyl-1,3-oxazol-5-yl] ace-tate	336.3413	3.7	0	5	6	73.9	25	8.927	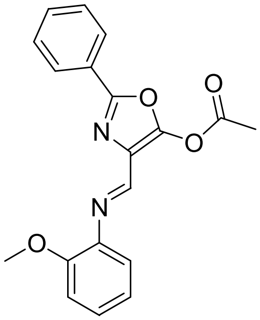
S21		[4-[(2-methoxy-phenyl)imi	350.3	4.2	0	5	7	73.9	26	39.987	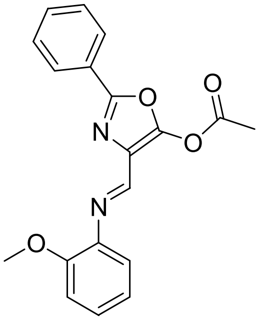

	SMR000039890	nomethyl]-2-phenyl-1,3-oxazol-5-yl] propanoate	679								
S22*	SMR000041595	[2-(furan-2-yl)-4-(phenyliminomethyl)-1,3-oxazol-5-yl] furan-2-carboxylate	348.3089	3.9	0	6	6	91	26	38.471	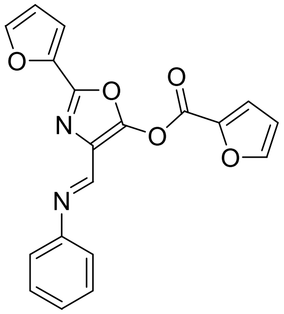
S23	SMR000046674	tert-butyl N-[(1R)-2-methyl-1-[5-[(3-methylphenyl)methylsulfonyl]-1,3,4-oxadiazol-2-yl]butyl]carbamate	423.5263	3.4	1	5	9	120	29	2.087	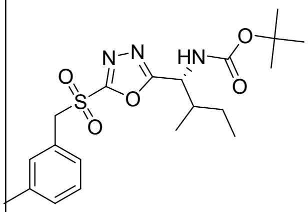
S24	ZINC00110897	2-[cyano-(4-methoxy-6-pyrrolidin-1-yl-1,3,5-triazin-2-yl)amino]acetamide	277.2825	0.7	1	3	5	121	20	14.196	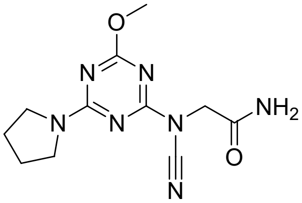

S25	T0509-1183	2-(4-chlorophenyl)sulfonyl-4,5-dimethyl-3,6-dihydrothiazine 1-oxide	319.8274	1.3	0	5	2	82	19	4.170	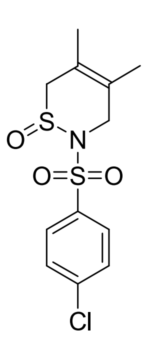
S26	MLS000049925	4-[1-[(5-methoxy-2-methylphenyl)methyl]benzimidazol-2-yl]-1,2,5-oxadiazol-3-amine	335.3598	2.5	1	4	4	92	25	37.190	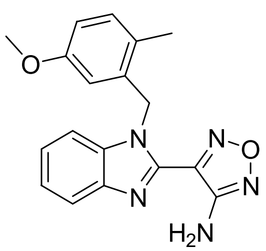
S27*	ST081935	4-[1-[(3-methoxyphenyl)methyl]benzimidazol-2-yl]-1,2,5-oxadiazol-3-amine	321.3333	2.2	1	4	4	92	24	45.971	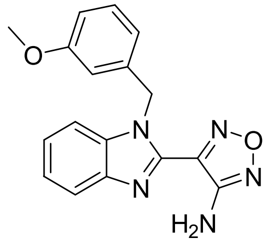
S28		benzotriazol-1-yl-(2-ethylsulfanylph	283.3	3.5	0	4	3	73.1	20	7.114	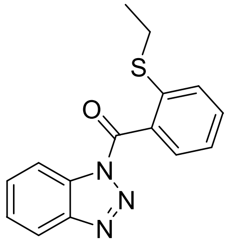

	MLS00 005013 8	enyl) meth- anone	48 2								
S29	SMR00 006160 5	1,8-dia- mino-3,6- dipyrroli- din-1-yl- 2,7-naph- thyridine- 4-carboni- trile	32 3.3 95 5	2.5	2	3	2	10 8	24	3.17 1	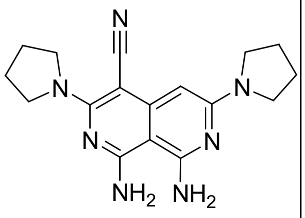
S30	AC1M MCYH	1,3-dime- thyl-5-phe- nyl-6- (1,2,4-tria- zol-4- yl)pyr- rolo[3,4- d]pyrimi- dine-2, 4-dione	32 2.3 21 3	0.8	0	2	2	76. 3	24	1.18 5	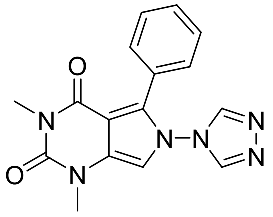
S31	SMR00 002157 2	[5-amino-1- (4-methox- y- phenyl)sul- fonylpyra- zol-3-yl] thiophene- 2-carbox- ylate	37 9.4 10 8	3.4	1	7	6	15 0	25	0.69 2	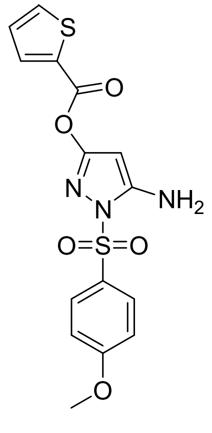

S32*	SMR000021568	[5-amino-1-(4-fluorophenyl)sulfonylpyrazol-3-yl] thiophene-2-carboxylate	367.3753	3.5	1	7	5	141	24	0.435	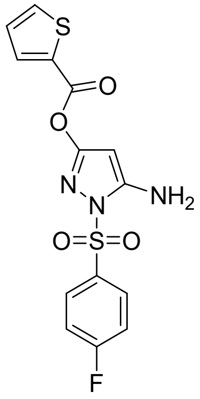
S33	ST081876	[3-oxo-2-(trifluoromethyl)-4H-1,4-benzoxazin-2-yl] acetate	275.1807	1.8	1	7	2	64.6	19	0.845	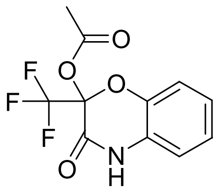
S34*	MLS000044152	[5-amino-1-(benzenesulfonyl)pyrazol-3-yl] thiophene-2-carboxylate	349.3848	3.4	1	6	5	141	23	0.247	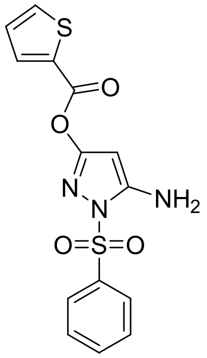

S35	STK382027	(1,3-dioxoisoindol-2-yl)methyl 2-(furan-2-carbonylamino)acetate	328.2762	1.3	1	6	6	106	24	8.563	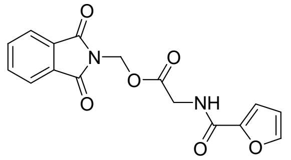
S36	MLS000110352	[2-(4-methoxyphenyl)-2-oxo-1-phenylethyl] 2-(furan-2-carbonylamino)acetate	393.3893	3.6	1	6	9	94.8	29	18.349	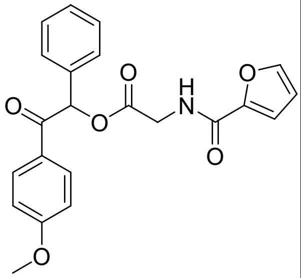
S37	SMR000037724	2-[[5,6-bis(furan-2-yl)-1,2,4-triazin-3-yl]sulfanyl]-N-phenylacetamide	378.4044	2.5	1	5	6	119	27	2.247	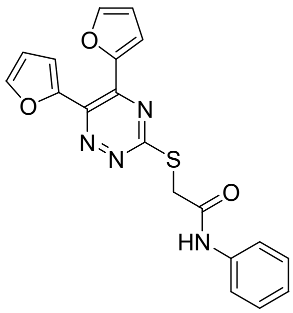
		methyl1-[3-methyl-2-[[3-									

S38*	CA-074Me (Cathepsin B Inhibitor IV)	(propylcarbamoyl)oxirane-2-carbonyl]amino]pentanoyl]pyrrolidine-2-carboxylate	397.4659	1.2	2	6	10	117	28	0.12	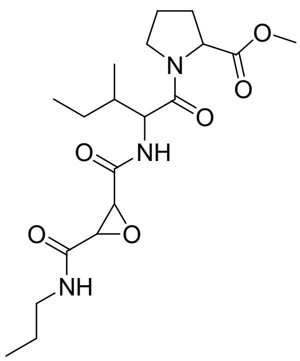
S39*	NCO 700	ethyl(2R,3R)-3-[[(2S)-4-methyl-1-oxo-1-[4-[(2,3,4-trimethoxyphenyl)methyl]piperazin-1-yl]pentan-2-yl]carbamoyl]oxirane-2-carboxylate; sulfuric acid	1141.2848	1.587	4	22	26	321	79	0.80	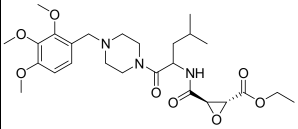

Table S2. *In silico* absorption, distribution, and toxicity prediction of natural compounds HIA: Human intestinal absorption; IVCCP: *in vitro* Caco-2 cell permeability; IVMCM: *in vitro* MDCK cell permeability; LogKp: *in vitro* skin permeability; IVPPB: *in vitro* plasma protein binding; BBP: *in vivo* blood-brain barrier penetration (C.brain/C.blood).

S.NO.	ABSORPTION				DISTRIBUTION		TOXICITY	
	HIA (%)	IVCCP (nm/sec)	IVMCM (nm/sec)	LogKp (cm/hour)	IVPPB (%)	BBP	Ames Test	Rodent Carcinogenicity
N1	13.061	20.375	0.406	-4.668	23.174	0.076	Negative	Positive
N2	9.607	20.803	0.225	-2.667	34.626	0.171	Positive	Positive
N3	94.509	7.509	10.103	-2.518	93.972	0.260	Negative	Positive
N4	96.427	21.254	0.076	-1.464	100.000	0.981	Negative	Positive
N5	40.026	20.705	0.394	-4.784	23.094	0.060	Positive	Negative
N6	93.609	18.352	7.489	-4.135	79.101	0.102	Negative	Negative
N7	65.273	20.915	0.044	-2.960	67.277	0.074	Negative	Negative
N8	3.963	20.622	0.607	-5.144	62.309	0.033	Negative	Negative
N9	40.447	20.916	1.561	-4.530	46.755	0.0399	Positive	Positive
N10	95.064	24.952	0.044	-2.711	91.424	0.676	Negative	Positive
N11	95.032	27.785	104.029	-3.618	86.798	10.117	Positive	Negative
N12	65.010	21.069	116.609	-3.463	63.956	0.046	Positive	Positive
N13	91.410	11.982	0.044	-2.843	95.553	0.147	Negative	Positive
N14	32.662	20.711	0.809	-4.493	28.793	0.049	Positive	Positive
N15	15.830	20.562	0.347	-4.611	30.875	0.078	Negative	Negative
N16	92.288	21.305	0.070	-3.551	79.143	0.056	Negative	Positive
N17	15.100	19.847	5.537	-2.796	56.532	0.066	Negative	Negative
N18	61.648	21.032	0.046	-2.804	59.853	0.039	Negative	Negative
N19	7.718	16.871	2.159	-2.424	52.798	0.054	Negative	Negative
N20	83.164	24.453	80.347	-3.496	72.145	0.087	Positive	Positive
N21	87.426	12.594	0.044	-3.390	100.000	0.155	Positive	Positive
N22	83.317	20.679	0.580	-4.407	10.104	0.106	Negative	Positive

Table S3. *In silico* absorption, distribution, and toxicity prediction of synthetic compounds. HIA: Human intestinal absorption; IVCCP: *in vitro* Caco-2 cell permeability; IVMCM: *in vitro* MDCK cell permeability; LogKp: *in vitro* skin permeability; IVPPB: *in vitro* plasma protein binding; BBP: *in vivo* blood-brain barrier penetration (C.brain/C.blood).

S.NO.	ABSORPTION				DISTRIBUTION		TOXICITY	
	HIA (%)	IVCCP (nm/sec)	IVMCM (nm/sec)	LogKp (cm/hour)	IVPPB (%)	BBP	Ames Test	Rodent Carcinogenicity
S1	60.194	20.463	0.678	-4.197	56.313	0.013	Negative	Positive
S2	97.100	17.117	6.729	-2.289	97.558	0.378	Negative	Negative
S3	93.609	18.352	7.489	-4.137	79.101	0.102	Positive	Positive
S4	97.889	9.923	250.199	-3.699	95.106	0.103	Positive	Positive
S5	95.283	42.293	95.284	-3.746	81.683	0.009	Negative	Negative
S6	77.733	21.220	0.616	-4.199	57.338	0.013	Negative	Negative
S7	60.194	20.463	0.678	-4.197	56.313	0.013	Negative	Positive
S8	72.657	16.881	0.024	-2.287	16.239	0.130	Positive	Negative
S9	97.495	18.261	0.517	-2.230	95.710	0.271	Negative	Negative
S10	91.484	13.392	25.177	-2.697	95.960	0.190	Negative	Negative
S11	95.717	20.117	10.424	-3.310	96.215	0.217	Positive	Positive
S12	95.960	11.466	3.549	-3.093	80.519	0.486	Negative	Negative
S13	63.994	17.806	38.07	-3.926	36.675	0.190	Positive	Negative
S14	93.357	7.063	21.422	-2.904	98.632	0.286	Negative	Negative
S15	92.130	28.608	4.571	-4.352	45.801	0.249	Negative	Negative
S16	95.736	9.338	46.659	-1.285	100.000	0.332	Negative	Positive
S17	96.969	19.128	13.666	-4.029	41.659	0.397	Positive	Negative
S18	94.125	9.974	139.033	-2.554	92.498	0.367	Negative	Negative
S19	98.873	47.116	1.352	-2.682	92.407	1.663	Positive	Positive
S20	94.552	8.701	6.193	-2.787	98.610	0.298	Negative	Negative
S21	97.487	11.641	213.774	-3.774	92.168	0.911	Positive	Positive
S22	95.176	10.491	7.823	-2.440	93.336	0.385	Negative	Negative
S23	98.738	44.202	111.905	-3.138	92.102	1.609	Positive	Positive
S24	97.765	30.127	0.666	-3.103	100.000	0.226	Positive	Positive
S25	98.275	36.071	69.342	-3.014	91.226	0.046	Positive	Negative
S26	97.895	9.023	210.895	-3.638	94.514	0.078	Positive	Positive
S27	59.805	20.520	1.116	-4.097	59.580	0.063	Negative	Positive
S28	89.959	4.343	1.303	-4.297	29.916	0.391	Positive	Positive
S29	95.082	3.020	51.153	-4.005	76.899	0.563	Positive	Negative
S30	98.945	21.835	6.399	-4.345	76.984	0.360	Positive	Positive
S31	92.965	13.529	4.761	-2.600	96.508	0.198	Negative	Negative
S32	98.926	43.493	57.630	-2.897	90.855	1.337	Positive	Positive
S33	95.032	27.785	104.029	-3.618	86.798	10.117	Positive	Negative
S34	97.107	23.644	115.936	-3.359	92.084	0.427	Positive	Positive
S35	93.359	20.672	161.295	-2.596	96.258	0.738	Positive	Negative
S36	96.051	15.071	205.377	-4.255	57.891	0.193	Positive	Positive
S37	61.374	20.691	1.996	-4.036	29.833	0.061	Negative	Negative
S38	96.051	15.071	205.377	-4.255	57.891	0.193	Positive	Positive
S39	77.733	21.220	0.616	-4.199	57.338	0.013	Negative	Positive

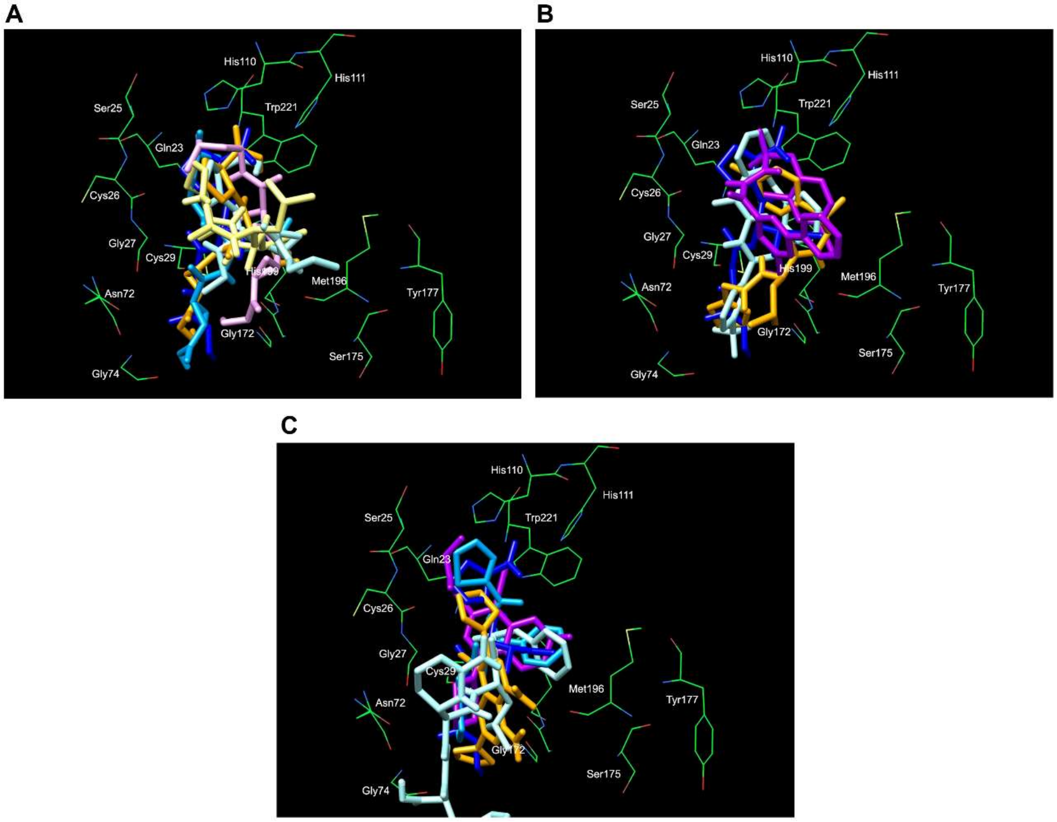

Figure S1. (A) Active site of Cathepsin B, displaying the binding mode of some Cathepsin B inhibitors, (B) virtual screening hits belonging to active cluster 2 compounds displaying π- π interactions and (C) virtual screening hits belonging to less active cluster 1 contains molecules with distinct binding mode. Some representatives of structures are shown.

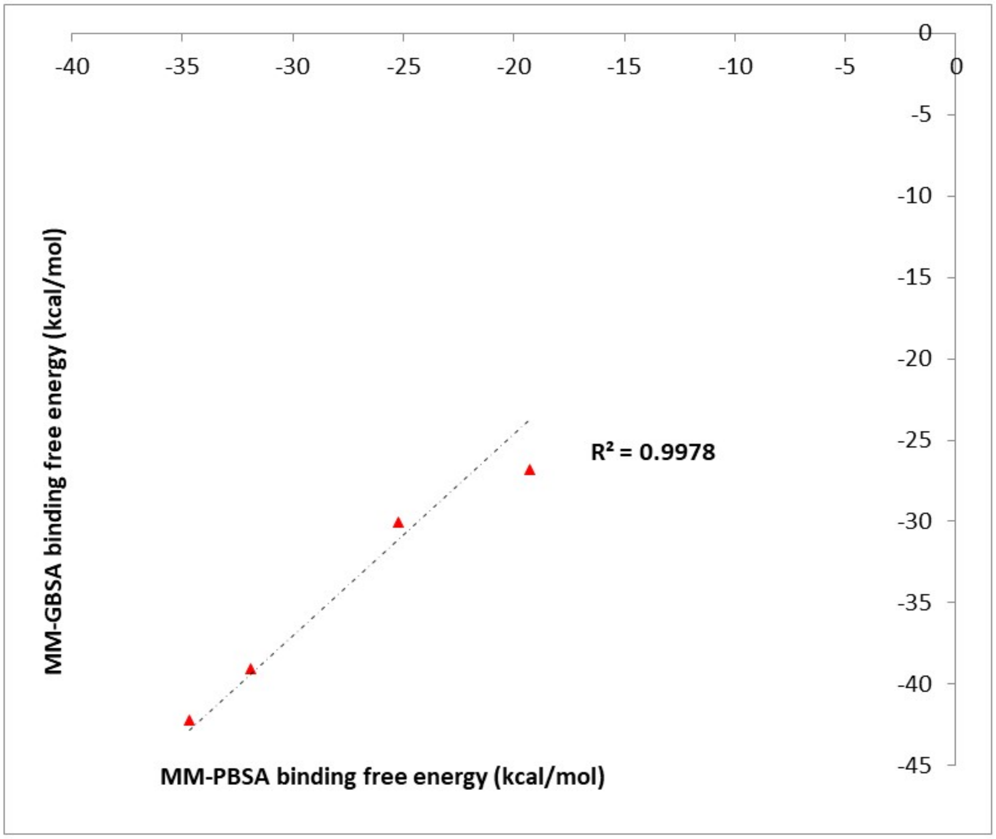

Figure S2. Correlation plot between MM-PBSA and MM-GBSA predicted binding free energy of in silico screening hits.
